# Large Variations in HIV-1 Viral Load Explained by Shifting-Mosaic Metapopulation Dynamics

**DOI:** 10.1371/journal.pbio.1002567

**Published:** 2016-10-05

**Authors:** Katrina A. Lythgoe, François Blanquart, Lorenzo Pellis, Christophe Fraser

**Affiliations:** 1 Department of Zoology, Tinbergen Building, University of Oxford, Oxford, United Kingdom; 2 Department of Infectious Disease Epidemiology, School of Public Health, Imperial College London, St. Mary’s Campus, London, United Kingdom; 3 Mathematics Institute, Zeeman Building, University of Warwick, Coventry, United Kingdom; 4 Big Data Institute, Li Ka Shing Centre for Health Information and Discovery, Nuffield Department of Medicine, University of Oxford, Oxford, United Kingdom; Princeton University, UNITED STATES

## Abstract

The viral population of HIV-1, like many pathogens that cause systemic infection, is structured and differentiated within the body. The dynamics of cellular immune trafficking through the blood and within compartments of the body has also received wide attention. Despite these advances, mathematical models, which are widely used to interpret and predict viral and immune dynamics in infection, typically treat the infected host as a well-mixed homogeneous environment. Here, we present mathematical, analytical, and computational results that demonstrate that consideration of the spatial structure of the viral population within the host radically alters predictions of previous models. We study the dynamics of virus replication and cytotoxic T lymphocytes (CTLs) within a metapopulation of spatially segregated patches, representing T cell areas connected by circulating blood and lymph. The dynamics of the system depend critically on the interaction between CTLs and infected cells at the within-patch level. We show that for a wide range of parameters, the system admits an unexpected outcome called the shifting-mosaic steady state. In this state, the whole body’s viral population is stable over time, but the equilibrium results from an underlying, highly dynamic process of local infection and clearance within T-cell centers. Notably, and in contrast to previous models, this new model can explain the large differences in set-point viral load (SPVL) observed between patients and their distribution, as well as the relatively low proportion of cells infected at any one time, and alters the predicted determinants of viral load variation.

## Abstract 2

A novel metapopulation model of HIV suggests that within-host infections are characterized by a highly dynamic process of localized infection followed by clearance within T cell centers.

## Author Summary

When a person is infected with HIV, the initial peak level of virus in the blood is usually very high before a lower, relatively stable level is reached and maintained for the duration of the chronic infection. This stable level is known as the set-point viral load (SPVL) and is associated with severity of infection. SPVL is also highly variable among patients, ranging from 100 to a million copies of the virus per mL of blood. The replicative capacity of the infecting virus and the strength of the immune response both influence SPVL. However, standard mathematical models show that variation in these two factors cannot easily reproduce the observed distribution of SPVL among patients. Standard models typically treat infected individuals as well-mixed systems, but in reality viral replication is localised in T-cell centres, or patches, found in secondary lymphoid tissue. To account for this population structure, we developed a carefully parameterised metapopulation model. We find the system can reach a steady state at which the viral load in the blood is relatively stable, representing SPVL, but surprisingly, the patches are highly dynamic, characterised by bursts of infection followed by elimination of virus due to localised host immune responses. Significantly, this model can reproduce the wide distribution of SPVLs found among infected individuals for realistic distributions of viral replicative capacity and strength of immune response. Our model can also be used in the future to understand other aspects of chronic HIV infection.

## Introduction

In 1979, Bormann and Likens introduced the concept of the shifting-mosaic steady state (SMSS) to describe biomass in forested ecosystems. This concept was based on the intuition that although the patches comprising the forested ecosystem might each be in different phases of ecological succession due to past disturbance events, the biomass of the whole forest will be at an equilibrium [1,2]. We suggest that for pathogens that cause systemic infection, such as HIV or hepatitis C virus, the viral population, host cells, and the immune system form a complex ecosystem within the host, with localized succession dynamics. We focus on HIV, characterized by rapid dynamics and trafficking between localized sites of replication in the body. The hypothesis that HIV is at SMSS in some individuals explains why viral loads vary so dramatically among patients, why only a small proportion of patients are natural controllers, and why a relatively low proportion of cells are infected during chronic infection.

Set-point viral load (SPVL) is the approximately constant viral load observed during early chronic asymptomatic infection. It varies by four orders of magnitude between patients [3] and is the most commonly used and robust predictor of the severity of infection [4,5]. Factors that have been implicated in determining SPVL include how rapidly the virus replicates and infects new cells [6–8], the efficacy of the cytotoxic T lymphocyte (CTL) immune response [9], and the activation rate of susceptible cells [10,11], all of which, in vivo, are probably influenced by a combination of viral and host factors [12,13]. However, using standard models of HIV within-host dynamics, in which the virus, susceptible and infected cells, and CTLs are assumed to be well mixed, these factors only mildly affect the SPVL unless the virus is close to extinction [14–17]. Introducing more complicated functions to describe the rate at which CTLs accumulate in response to the number of infected cells can help to explain more of the variation in SPVL [18–22], as can small differences in a large number of parameters [20]. Even with these models, though, it is still hard to explain orders of magnitude differences in SPVLs without fine-tuning parameters, and especially to reproduce the left tail of the distribution composed of patients with low viral loads. As a further refinement to these models, incorporating activation of cells from a viral reservoir can explain very low viral loads (below the level of detection by conventional assays) for parameters in which otherwise the virus is expected to go extinct, e.g., [22,23], although not the large number of patients with low viral loads but above the level of detection.

HIV replication is focused in the secondary lymphoid organs, such as the lymph nodes (LNs), the spleen, and the gut [24]. At an even finer scale, replication is likely centered within the T cell areas of these organs, such as the Malpighian bodies found in the spleen [25] and Peyer’s patches in the gut [24]. Genetic analysis has revealed that the genetic composition of the viral population differs among these sites of replication, even at very fine scales [25,26], and that this differentiation might well be transient [27]. This has led to the speculation that the within-host structure of HIV is best thought of as a metapopulation, in which T cell areas can be considered patches of HIV replication, and with long-range migration of the virus among these patches via the blood [28–30]. This differentiation is formally supported by calculations of F_ST_ (a measure of the genetic differentiation among groups or patches) from viruses in the spleen, which reveals high levels of differentiation in most patients and is suggestive of a within-host metapopulation structure [30].

Here, we develop a metapopulation model of within-host HIV dynamics during chronic infection in which within-patch dynamics are explicitly incorporated, and which we investigate in the form of population-based stochastic simulations. To gain further analytical insight, we also derive a mathematically tractable analytical approximation in which within-patch dynamics are nested within a model of between-patch dynamics, using a time-since-infection framework to link the two scales [31,32]. We show that for a wide range of parameters, the system as a whole reaches a steady state even though the patches themselves are not necessarily at equilibrium. The dynamics of the system at the whole patient (between-patch) level depend critically on the interaction between CTLs and infected cells at the within-patch level. If the CTL response is able to clear infected cells from patches before a within-patch steady state is established, the metapopulation can reach a dynamic SMSS, at which the whole body’s viral population is stable over time but the overall equilibrium results from an underlying, highly dynamic process of local infection and clearance within T-cell centers. If the CTL response is not able to clear infected cells from patches, the system will reach a full equilibrium (FE), at which all of the patches, as well as the system as a whole, are at equilibrium. In contrast to the corresponding single-patch model, the metapopulation model can explain the distribution of SPVLs observed among patients due to modest differences in viral infectivity and the strength of the CTL response without model fine-tuning.

## Results

**Fig 1:**
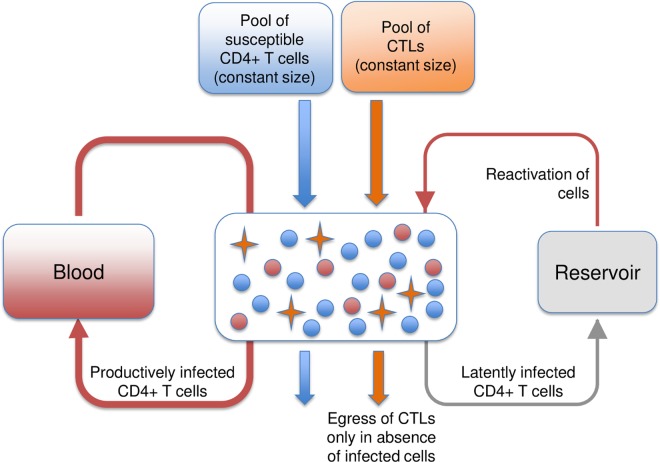
The trafficking of lymphocytes through a patch. Susceptible CD4+ T cells, infected CD4+ T cells, and cytotoxic T lymphocyte (CTL) cells continually traffic through the patches in the metapopulation. We assume a constant input of susceptible cells and HIV-specific CTLs from the blood into the patches and therefore that CD4+ T cell production and CTL production are able to compensate for any losses. In contrast, the inflow of infected cells into a patch is directly proportional to the total number of infected cells in the blood plus, if present, the number of latently infected resting CD4+ T cells in the reservoir; immediately after being infected by the virus, a small proportion of CD4+ T cells enter into a resting state, thus becoming part of the reservoir, and reenter general circulation when reactivated [34,35]. Since little is known about the trafficking of latently infected resting CD4+ T cells, upon reactivation, we make the simplifying assumption that these cells directly enter patches. Infected and susceptible CD4+ T cells exit patches at the same per capita rate, as do CTLs in the absence of infected cells. However, if infected CD4+ T cells are present, egress of HIV-specific CTLs (but not other CTLs) is prevented, resulting in their gradual accumulation due to the continued immigration of CTLs from the blood. As HIV-specific CTLs accumulate, the rate at which infected cells are killed due to the CTL response increases, and if this rate of killing is sufficiently high, infected cells will eventually be eliminated from the patch and, subsequently, egress of HIV-specific CTLs from the patch will resume. Blue circles represent susceptible cells, orange stars represent HIV-specific CTLs, and red circles represent infected cells.

In our model of HIV chronic infection, we assume viral replication is centered in a large number of discrete patches, representing T-cell zones within the secondary lymphoid tissues (Fig 1). Susceptible CD4+ T cells, infected CD4+ T cells, and HIV-specific CTLs (which target infected CD4+ T cells) continuously traffic through these patches via the blood, with the accumulation of HIV-specific CTLs within patches when infected cells are present. As the number of CTLs increases, so does the rate at which infected cells are killed, until a maximum rate of killing is reached. Because the presence of a reservoir of latently infected, long-lived resting CD4+ T cells is well established [33] and can explain very low viral loads, e.g., [22,23], we also include a reservoir in the model to investigate the impact this might have on the dynamics of HIV infection. The model is described by Eqs 1.1–1.5 (Methods) and is parameterised using detailed observational data (Methods and Table 1).

**Table 1:** Model parameters.

*N*	Number of patches	10,000	See Methods
*M*	Per capita rate at which CD4+ T cells leave the blood and enter patches	48 per day	[36]
*x*_*B*_	Number of susceptible cells in the blood	1.67 x 10^7^	See Methods
*γ*_*i*_	Probability that a cell leaving the blood or reactivating from the latent reservoir enters patch *i*	1/N ∀ *i*	
*β*_*i*_	Per capita infectivity in patch *i,* $\bar{\beta }=\sum_{i}\beta_{i}/N$	Variable [1–20] per day	See Methods
*d*	Death rate of susceptible cells	0.01 per day	[37,38]
*ε*	Rate that lymphocytes exit patches	2.5 per day	[39,40]
$x_{i}^{max}$	Number of susceptible cells in a patch in the absence of infection, $x_{i}^{max}=\gamma_{i}\;M\;x_{B}/\left(d+\varepsilon \right)$	3.2 x 10^8^/N	See Methods
*λ*	Proportion of CD4+ T cells that enter a resting state upon infection	0.001	[34,35]
*a*	Activation rate of latently-infected resting CD4+ T cells	0.001 per day	[34,35]
*ω*	Proportion of activated resting CD4+ T cells that produce infectious virions	0.01	[41]
*δ*	Death rate of infected cells within patches	1 per day	[42,43]
*δ*_*B*_	Death rate of infected cells in the blood	1 per day	[42,43]
*M*_*e*_	Effective migration rate, *M*_*e*_ = *ε M*/(*M* + *δ*_*B*_)	2.4 per day	
*k*	Maximum rate that CTLs kill infected cells within a patch	Variable [0–20] per day	See Methods
*z*^*max*^	Maximum number of CTLs that a patch can accommodate	1x10^11^/N	See Methods
*c*	CTL immigration rate constant	0.5 per day	See Methods
*δ*_*L*_	Death rate of latently infected resting CD4+ T cells	0.0005 per day	[34]
$\bar{L}$	Fixed reservoir size (number of latently infected CD4+ T cells) assumed in analytical approximation and single patch model	0 or 10^7^	[44]
*p*	Production rate of virions in the blood	5x10^4^ per day	[45]
*r*	Removal rate of virions from the blood	23 per day	[46]
*Vol*_*B*_	Volume of blood in a typical human adult	5000ml	

All values assume “high” *M*_*e*_

We investigated the behavior of the model for differing values of viral infectivity, 1 ≤ *β* ≤ 20 per day, and the maximum rate at which HIV-specific CTLs kill infected cells within a patch, 0 ≤ *k* ≤ 20 per day (see Methods). These two factors are associated with variation in SPVL among patients and are reasonably expected to vary among individuals due to both host and viral factors [6–9,12]. We also investigated three values of the “effective migration rate” *M*_*e*_, which describe the rates at which the cells transit between the patches via the blood.

### Dynamical Behavior of the Metapopulation Model

**Fig 2:**
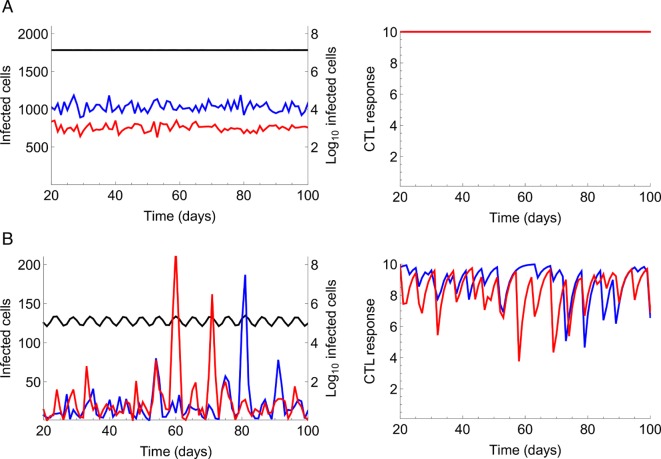
Examples of simulation dynamics. (A) Full equilibrium (FE). (B) Shifting mosaic steady state (SMSS). A total of 10,000 patches were simulated, with the left panel showing the number of infected cells in two of these patches (red and blue lines, left-axis) and the total number of infected cells in the whole metapopulation, summed over all the patches and the blood, but not the reservoir (black line, right-axis; note this is on a Log_10_ scale). The right panel shows the CTL response, measured as the rate at which CTLs kill infected cells in each of these two patches, which is proportional to the number of CTLs in the patches. (A) Viral infectivity, $\bar{\beta }$ = 13 per day, maximum strength of the CTL response, *k* = 10 per day. (B) $\bar{\beta }$ = 8 per day, *k* = 10 per day. We assumed a high effective migration rate (*M*_*e*_ = 2.4 per day). The simulation was initiated with 10^8^ infected cells randomly distributed among the patches and no reservoir. See Table 1 for all other parameters.

To investigate the behavior of the model, we first developed a simulation based on a stochastic counterpart of the set of ordinary differential equations that describe the metapopulation model (Eq 1.1–1.5). To accommodate heterogeneity among patches, for each simulation, we sampled viral infectivity for each patch from a uniform distribution with mean $\bar{\beta }$ in the range 0.5$\bar{\beta }$–1.5$\bar{\beta }$, thus implicitly assuming some patches are more densely packed with T cells than others (see Methods, S1 Simulation Code, and S1 Data). Depending on $\bar{\beta }$, and the maximum strength of the immune response, *k*, the dynamics of the metapopulation typically converges to one of three stationary states: a disease-free trivial stationary state, a “full equilibrium” (FE) at which the patches and the metapopulation as a whole are at a stationary state (Fig 2A), and an SMSS in which the number of infected cells and HIV-specific CTLs in some, or all, of the patches fluctuates, but the total number of infected cells is relatively constant (Fig 2B). To gain a sense of the dynamics of the model when at SMSS, we have provided an animation (S1 Animation).

### Insights from the Analytical Approximation to the Metapopulation Model

**Fig 3:**
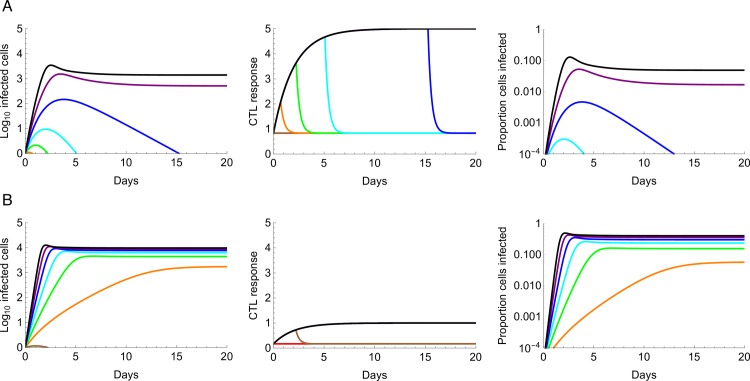
Examples of within-patch dynamics for a single colonization event used for the analytical approximation. (A) Maximum strength of the CTL response, *k* = 5 per day. For high levels of viral infectivity *β =* 9, 10 per day (purple and black lines, respectively), an initial burst of infection is followed by a long-term endemic state. The CTL response is the rate at which CTLs kill infected cells, which is proportional to the number of HIV-specific CTLs in the patch. This increases due to the prevention of egress of HIV-specific CTLs, but even when the maximum rate of CTL killing, *k*, is reached, this is not sufficient to clear the infected cells from the patch. Even though levels of viral infectivity are high, only a small proportion of cells are infected. For moderate levels of infectivity, *β* = 5, 6, 7, 8 per day (orange, green, cyan, and blue lines, respectively), an initial burst of infection also occurs, accompanied by an increase in the strength of the CTL response. Eventually, the rate of CTL killing becomes sufficiently high to eliminate the infected cells from the patch, and subsequently the HIV-specific CTLs egress from the patch. For lower levels of infectivity, bursts of infection fail to establish. (B) Maximum strength of the CTL response, *k* = 1 per day. For moderate and high levels of viral infectivity (*β* = 5, 6, 7, 8, 9, 10 per day), an endemic state is now reached, and a much higher proportion of cells are infected compared to when *k* = 5 per day. Equations describing the within-patch dynamics are found in S1 Text, and model parameters are as described in Table 1.

Although analytical expressions for the disease-free equilibrium and FE of the metapopulation model can be found directly from the differential equations, this is not possible for the SMSS. We derived a deterministic, mathematically tractable approximation to the full metapopulation model, assuming all patches are identical, using a time-since-infection framework (see S1 Text). Which of the three metapopulation steady states is established, disease-free, FE, or SMSS can be understood by considering the within-patch dynamics (Fig 3). When an infected cell entering a patch fails to establish a local burst of infection, the metapopulation is at the disease-free equilibrium. When an infected cell establishes a non-zero endemic equilibrium in a patch, the metapopulation is at FE. When an infected cell establishes a local burst of infection, which is then cleared by the CTL response, the metapopulation will be at SMSS if these local bursts can propagate from patch to patch and sustain the infection. In mathematical terms, the “patch-to-patch” reproduction number (*R*_*P*_), the number of patches that are colonized as a result of a typical single burst in an otherwise fully susceptible host, must be greater than one (otherwise disease-free equilibrium occurs). This is analogous to the concept of *R*_*0*_ in epidemiology, where an epidemic will not be sustained if the number of people that a typical person infects, in a totally susceptible population, is less than one [47]. Which of these equilibria occurs depends critically on the infectivity of the virus, *β*, and the maximum strength of the CTL response, *k* (see S1 Text for details).

As an additional observation, the proportion of susceptible cells that are infected within a patch is low for most of the parameter values we tested (often less than 5%, Fig 3A), in agreement with what is observed at sites of HIV replication during chronic infection [48]. This is in contrast to most well-mixed models, in which almost all susceptible cells are expected to be infected (although see [19] for an exception). This disparity occurs because of the high rates at which CD4+ T cells traffic through the patch (with a mean residence time of about 10 hours), and because there is insufficient time to infect a large number of susceptible cells when within-patch bursts of infection are short lived. During acute infection, a much larger proportion of CD4+ T cells tend to be infected than in chronic infection (up to 60% of memory CD4+ T cells [49,50]), probably because the full force of the host immune system is yet to kick in [49]. In agreement with this observation, if we set the immune response in our model to be very weak (*k* = 1 per day; see Fig 3B), the CTL response fails to control the viral infection and about half of the activated cells in a patch will be infected (unless the infectivity of the virus is extremely low). Because in this low immunity scenario the viral population is target-cell limited, once the within-patch dynamics have reached equilibrium we see little variation in the proportion of cells that are infected for large differences in viral infectivity (*β*).

It is also interesting to note, from Fig 3, that although the maximum strength of the CTL response in a patch, *k*, might be relatively high, the actual rate at which CTLs kill infected cells (measured as *k* * *z*(*τ*)/*z*^*max*^, where *z*(*τ*) is the number of HIV-specific CTLs in the patch at time *τ* since the patch was infected, and *z*^*max*^ is the maximum possible number of HIV-specific CTLs the patch can hold) is often much lower. This might, in part, explain the large discrepancy between different estimates of the rate at which CTLs kill infected cells (see Methods).

### Number of Infected Cells during Chronic Infection

We next assessed the impact that a metapopulation structure has on the number of infected cells within an infected individual during chronic infection, which is assumed to be the stationary state number of infected cells in our models. Specifically, we compared the metapopulation simulation model with the analytical approximation of this model and the equivalent single-patch differential equation model in which lymphocytes are assumed to traffic between one well-mixed patch and the blood (see Methods).

**Fig 4:**
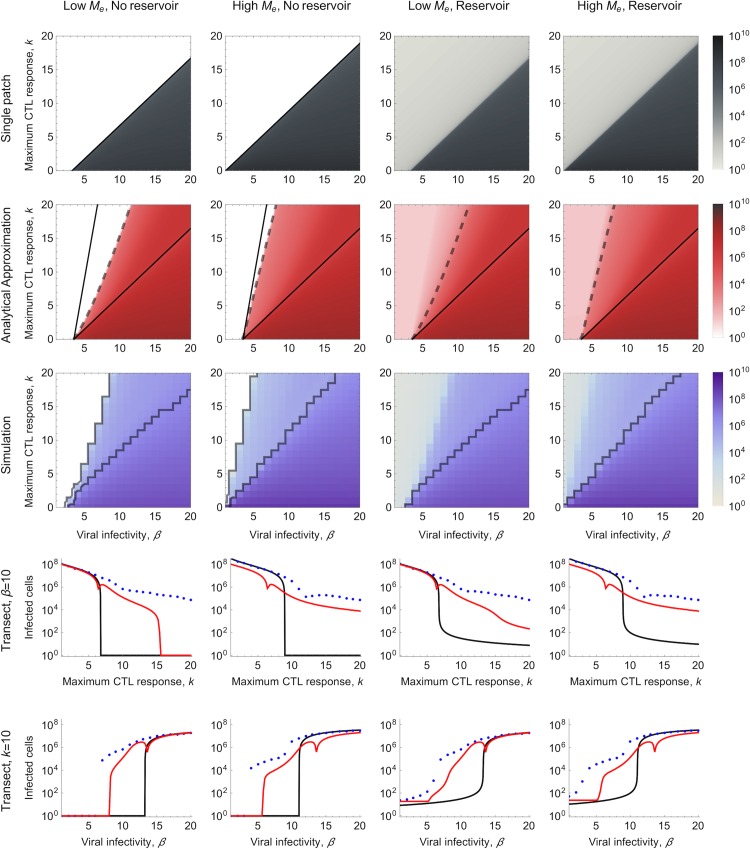
Total number of infected cells for varying viral infectivity, *β*, and maximum CTL response, *k*. The density plots in the top three rows show the number of infected cells at stationary state for different *β* and *k* (note that in the simulations, mean viral infectivity varies across patches, and therefore mean viral infectivity $\bar{\beta }=\beta$ is plotted). Results are shown without and with a reservoir included in the model, and for a low effective migration rate (*M*_*e*_ = 0.25 per day, *δ*_*B*_ = 432 per day) and a high effective migration rates (*M*_*e*_ = 2.4 per day, *δ*_*B*_ = 1 per day). For the single patch model, the black line shows the extinction threshold above which the viral population cannot be sustained in the absence of a reservoir. For the analytical approximation, the area between the solid black lines shows where introduction of infection in an uncolonised patch is followed by a within-patch burst, and the dashed line indicates where the patch reproduction number *R*_*P*_ = 1, thus distinguishing whether, in the absence of a reservoir, the virus goes extinct or an SMSS can be sustained. To the right of the SMSS region, a full equilibrium (FE) is predicted, and to the left, a trivial equilibrium exists. The areas in white are those where no infected cells are present. For the simulation, the area between the black solid lines shows where an SMSS is observed, defined as where both the viral population has not gone extinct and the mean CTL response across all patches is less than 99% of the maximum CTL response. The bottom two rows are transects across the density plots shown above them, with either viral infectivity held constant (*β* = 10 per day) or the maximum strength of the CTL response held constant (*k* = 10 per day). Single patch model, grey-scale gradient and back transect line; analytical approximation of the metapopulation model, red-scale gradient and red transect line; simulation of the metapopulation model, blue-scale gradient and blue transect dots. All other parameters are as described in Table 1.

We first consider the total number of infected cells for different values of viral infectivity, *β*, and the maximum strength of the CTL response, *k*, in the absence of a reservoir of latently-infected resting CD4+ T cells (Fig 4, columns 1 and 2). Significantly, for a large range of parameters, spatial structure enables the viral population to persist at an SMSS in the metapopulation model, whereas extinction is predicted in the single-patch model for the same parameters (Fig 4). Moreover, when the metapopulation is at SMSS, a broad range in the number of infected cells is predicted for relatively small changes in *β* and *k*. In contrast, the single-patch model exhibits strong threshold-like behavior, where the number of infected cells is typically either zero (low *β*, high *k*) or very high (high *β*, low *k*), with intermediate viral loads only possible when the parameters are close to the virus “extinction threshold” [15]. Note that we have presented results for both low and high effective migration rates (*M*_*e*_) among patches. The low *M*_*e*_ scenario is used to check the analytical approximation, because it most closely reflects the assumption of no patch super-infection required for the analytical approximation (achieved by increasing the death rate in the blood from a realistic *δ*_*B*_ = 1 per day to *δ*_*B*_ = 432 per day; see Methods). The high *M*_*e*_ scenario reflects estimates of lymphocyte trafficking made from experimental data (see Methods and Table 1).

Wide variations in the number of infected cells occur at SMSS because colonized patches are, for most of the time, in a near-exponentially growing phase (Fig 2). Thus, modest differences in viral infectivity and the strength of the CTL response translate into very large differences in the total number of cells infected during a burst of infection. In other words, when the system is at FE, the virus is close to carrying capacity within each of the patches, and is therefore sensitive to parameter values that affect this carrying capacity (e.g., the rate at which susceptible cells enter patches). However, at SMSS, the system becomes much more sensitive to parameter values that affect the rate at which the number of infected cells and, by proxy, the viral population grows (e.g., *β* and *k*).

If a reservoir is present (Fig 4, columns 3 and 4), the same qualitative arguments apply, although the virus does not go extinct (at least in the short term) because it is maintained at very low levels by the reactivation of cells from the reservoir. In the parameter range in which local bursts of infection are possible but *R*_*P*_ is less than one, more infected cells are maintained in the metapopulation compared to the single-patch model, in which localized bursts of infection are not expected. This has similarities to the verbal argument proposed by Grossman et al. [28], who suggested that a reservoir is needed for HIV to persist within hosts at low SPVLs.

The wide variations in the number of infected cells observed in the metapopulation stationary state are robust to the key unknowns in the model, specifically the number of patches (S1 and S2 Figs) and the effective migration rate (*M*_*e*_; S3 and S4 Figs), although, as expected, the threshold-like behavior of the model increases if *M*_*e*_ is substantially increased or if the number of patches decreases. Due to uncertainties in how HIV-specific CTLs accumulate in patches, we also analysed an alternative to the “CTL immigration” metapopulation model described here, which we call the “CTL proliferation” metapopulation model. In this alternative version of the model, CTLs accumulate within patches due to local proliferation rather than the immigration of CTLs from outside of the patch (see S1 Text and S5–S9 Figs). Although this also has the effect of increasing the threshold-like behavior of the model, a broad range in viral loads is still observed, making our conclusions robust to this fundamental change in the modeling assumptions. It is noticeable from the simulation dynamics (Fig 2B) that at SMSS the number of infected cells at the population level can oscillate, suggesting the dynamics among patches are synchronized. However, the level of synchrony among patches is generally quite modest, as is the amplitude of the oscillations (S1–S4, S6–S9 Figs; see S1 Text for further discussion). Although with increasing patch heterogeneity these oscillations are likely to be eroded, it is tempting to speculate that differences in viral load measurements taken during untreated chronic infection for individual patients might partly reflect these dynamics.

### Testable Predictions

We have presented a model of the within-host metapopulation structure of HIV during untreated chronic infection. Conceptual models such as this should aid understanding, but also make qualitative and/or quantitative testable predictions that enable discrimination from other plausible models; in this case, the single patch model. Here, we outline four testable predictions of the metapopulation model: two of them are supported by available data, while the other two require further data collection and analysis to be tested.

(i) Distribution of set-point viral loads among individuals in a population

**Fig 5:**
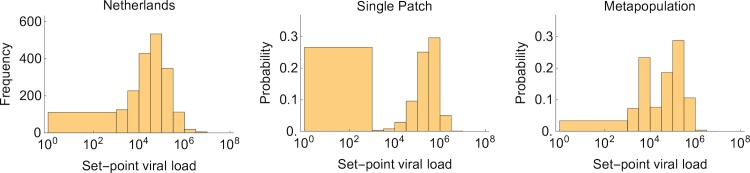
Predicted distributions of set-point viral load (SPVL) among a cohort of patients. (A) Histogram of the observed frequencies of SPVL among seroconverters in the Netherlands (S1 Table). Because of differences in the sensitivities of viral load tests, we pooled all individuals with a viral load less than 10^3^ per ml. (B) Maximum log-likelihood distribution of SPVLs for the single patch model given the Netherlands seroconverter data, with log $L(\mu_{\beta }=14,\sigma_{\beta }=4,\mu_{k}=15,\sigma_{k}=3|Data)$ = -5386. (C) Maximum log-likelihood distribution of SPVLs for the 10,000 patch metapopulation model given the Netherlands seroconverter data, with log $L(\mu_{\beta }=14,\sigma_{\beta }=4,\mu_{k}=15,\sigma_{k}=3|Data)$ = -4,013. For (B) and (C), viral infectivity, *β*, and the maximum CTL immune response, *k*, are assumed to be distributed according to truncated normal distributions, with probability density functions *f* (*β*; *μ*_*β*_,*σ*_*β*_,*β*_*min*_,*β*_*max*_) and *f* (*k*; *μ*_*k*_,*σ*_*k*_,*k*_*min*_,*k*_*max*_), respectively, where *β*_*min*_ = 1.05 per day, *k*_*min*_ = 0 per day, and *β*_*max*_ = *k*_*max*_ = 20 per day (see S1 Text). For both the metapopulation and single patch models, a viral reservoir is assumed to be present and the effective migration rate is assumed to be high (*M*_*e*_ = 2.4 per day). All other parameters are as described in Table 1.

Because this is a model of chronic infection, the most pertinent clinical data to test the model is the distribution of SPVLs observed among individuals. We fitted the metapopulation simulation and the corresponding single patch deterministic model to the distribution of patient SPVL measured amongst seroconverters in the Netherlands (S1 Table). Using the quasi-steady state assumption that the number of virions is proportional to the number of infected cells [16], and using the same reasoning as [45], we calculated the SPVL predicted by our models as approximately $y_{B}^{*}(\frac{p}{r*{Vol}_{B}})$, where $y_{B}^{*}$ is the mean number of infected cells in the blood at stationary state, *p* is the production rate of virions from infected cells (5x10^4^ per day [45]), *r* is the removal rate of virions from the blood (23 per day [46]), and *Vol*_*B*_ is the volume of blood in a typical human adult (5 litres). Since data on the distribution of *β* and *k* among patients is lacking, we assumed truncated normal distributions (see S1 Text for full details). For each set of parameters describing the distribution of *β* and k, the likelihood was computed as the product, over all SPVL values in the data, of the probability this SPVL value occurred under the model (S2 Data). The distribution of the Netherlands data and the maximum likelihood distributions for the single patch and metapopulation models are shown in Fig 5, and the bivariate marginal likelihood profiles are shown in S10 Fig.

For all distributions of *β* and *k* that we examined, the likelihood of the metapopulation model is considerably higher than that of the single patch model (S10 Fig). The poor fit of the single patch model is largely due to the large number of natural controllers it predicts (which we have defined as patients with SPVLs <1,000 per ml). Among the Netherlands seroconverters, 5.8% of patients are natural controllers. The maximum likelihood metapopulation model predicts 3.3% of patients should be natural controllers, whereas the maximum likelihood single patch model predicts 27% should be natural controllers. Additionally, the bivariate marginal likelihood surfaces are much flatter for the metapopulation compared to the single patch model, and therefore the metapopulation is much more robust to uncertainties in the true distributions of *β* and *k* (since high likelihood values are observed for a broad range of distributions). Although the metapopulation model is a much better fit to the data and captures well the left-tail in the distribution of SPVLs, it predicts a bimodal distribution that is not evident in the data, suggesting there are aspects of the biology that our model is not yet capturing, including heterogeneity among patches within a patient [40], non-normal and/or non-independent distributions of *β* and *k* among patients, and the role of other host and viral factors. Interestingly, the alternative CTL proliferation version of the model has the largest maximum likelihood value of all the models considered and does not predict a high proportion of natural controllers or a strong bimodal distribution, although the bivariate marginal likelihood surfaces are less flat than for the CTL immigration model (S10 Fig). Given these differences, future work should focus on which mechanism(s) best describes how CTLs accumulate within patches.

(ii) Distribution of infected cells within individuals

A prediction of the metapopulation model is that the number of infected cells should be unevenly distributed among potential sites of HIV replication at any one moment in time when at SMSS. The distribution of HIV in human spleens shows this pattern [25,51]. Additionally, observation of the spleens of simian immunodeficiency virus (SIV)-infected macaques [52] shows that virus with high replicative capacity is found in most, if not all, potential sites of replication (i.e., consistent with a FE), but that virus of low replicative capacity is not (i.e., consistent with SMSS). More direct evidence might come from real-time observation of individual sites of potential infection in vivo, from which we would predict the number of infected cells to increase and decrease in a succession of local epidemics intermittently controlled by the immune system, but this data is so far unavailable.

(iii) Genetic structure of the viral population within individuals

Previous studies have shown that the effective population size, *N*_*e*_, of HIV is much lower than consensus population size [53,54], and that *F*_*st*_ values (a measure of population subdivision) are high in the spleen, with values ranging from 0.08 to 0.6 in different patients [30]. These data provide strong evidence that HIV exhibits metapopulation dynamics that are characterised by colonisation and extinction of virus in potential sites of replication [30]; the same dynamics seen in the metapopulation model at SMSS. To formally test whether the metapopulation model can explain observed *N*_*e*_ and *F*_*ST*_ values, it will be necessary to extend the model to include explicit evolutionary processes, an analysis that will be a focus of future work. Another feature of HIV infections are the ladder-like time-resolved phylogenies observed during the course of infection [55,56]. The metapopulation model could be further extended to test whether phylogenies predicted by the model are different from those predicted from a single patch model, and whether these more closely resemble observed phylogenies.

(iv) The dynamics of chronic infection

A prediction of the metapopulation model is that we might expect to see oscillations in viral load over short time-scales in patients with intermediate SPVLs. Testing this prediction would require obtaining viral load data from very frequently sampled individuals during chronic infection (every 1 or 2 weeks) and testing statistically for periodic oscillations. Unfortunately, suitable data is not yet publicly available. If these oscillations were found, it would be strong evidence for an SMSS type process, but we note that the absence of oscillations would not be strong evidence against SMSS; the within-host environment is likely to be much more heterogeneous than we have modelled, which would dampen out oscillations, and stochastic effects and errors in viral load estimates would mask any underlying oscillations.

Note that our model does not generate testable predictions regarding early infection dynamics, as it does not include biological features that are likely prominent in that phase, such as HIV-specific CTL accumulation, CD4+ T cell loss at the systemic level, and evolution of CTL escape. Similarly, our model does not generate testable predictions on the impact of antiretroviral therapy (ART), since both in the metapopulation and single patch models the viral load decline is rapid and driven by the death of infected cells, which is unaffected by patch structure, and by CTL killing (simulation not shown). Furthermore, for both models, the rate of viral load decline is much faster than observed in patients unless very low rates of CTL killing (*k* << 1 per day) are assumed, although it has been shown that adding an eclipse phase to single patch models can reconcile high values of *k* with the data [57].

## Discussion

We have developed a within-host metapopulation model of chronic HIV infection in which viral replication is focused within T cell centers, derived a mathematically tractable approximation, and ran stochastic simulations. For a broad range of parameters, the virus can be maintained at equilibrium even though the patches themselves are not at equilibrium. This quasi-equilibrium represents an SMSS, a concept first described 35 years ago by Bormann and Likens [1] to describe the biomass in forested ecosystems. Importantly, this model can easily account for the broad distribution of SPVLs observed among patients and the low proportion of susceptible cells that are infected during chronic infection due to differences in the replicative capacity of the virus (because of either host or viral factors) and the strength of the CTL response. Moreover, the model can explain why only a small number of patients (about 5%) go on to control the virus in the absence of therapy (<1000 virions per ml) after the initial burst of viremia during acute infection.

As well as explaining the wide range of viral loads observed among patients during chronic infection, our model also gives insight into the marked changes in viral load seen between acute, chronic, and late-stage infection. Around the peak of acute infection, when host immune responses against the virus are still weak [49], or during late stage infection, when the immune system has essentially collapsed, we suggest that within-patient dynamics are probably close to FE. The small impact of viral replication rate on FE viral load might explain why peak viremia tends to be similar among patients and why a large proportion of CD4+ T cells become infected with virus [49,50]. In contrast, during chronic infection, immune responses against the virus are much more effective; thus, we propose a large proportion of patients will be at an SMSS, with small differences in the strength of the host immune response and the infectivity of the virus explaining the broad ranges in viral loads. It is important to recognize that the metapopulation model we have presented here has been used to describe the chronic phase of HIV infection, during which, unlike during acute and late stage infection, the system can be assumed to be at a steady state. To fully test how a metapopulation structure might affect the predicted dynamics of the model over the entire course of the infection, and to fit it against early and late infection clinical data, would require further processes, such as HIV-specific CTL accumulation, CD4+ T cell loss at the systemic level, or evolution of CTL escape, to be modeled mechanistically or, at least, as external driving factors carefully parameterized from data. In both cases, incomplete biological knowledge would likely require strong assumptions to be made in parameter values and model structure.

In terms of caveats, we should also recognize that the proliferation and trafficking of lymphocytes around the body via the blood, lymph, secondary lymphoid tissues, and nonlymphoid tissues is complex and incompletely understood [36,58]. This matters because the dynamics of multitrophic systems can depend crucially on the precise assumptions used to formulate the model [19], and the within-patch processes modelled here are no different. Despite these gaps in our knowledge, the observations that not all T cell zones contain infected cells [51], that HIV is spatially and genetically structured in the body over very small spatial scales [25,26,52], that this structure might not be stable over time [27], that higher fitness clones tend to co-localise but lower fitness clones do not [52], the small effective population size of HIV compared to the actual population size [53,54], and high values of F_ST_ [30] together provide compelling evidence that the HIV within-host landscape is highly dynamic and characterised by local bursts of viral replication.

Although we have focused here on HIV, it is likely that consideration of within-patient spatial structure will also have implications for other chronic viruses, such as Hepatitis C, in which infection of hepatocytes in the liver is spatially structured [59], and where the number of hepatocytes infected correlates with viral load [60]. More generally, we think this model is useful in explaining immunopathogenesis, because it naturally explains large variations in viral load. It is also likely to be useful for studying viral evolution, and because it alters the predicted determinants of viral load to viral and host factors (*β* and *k*), it will lead to different evolutionary predictions. In a metapopulation that is at SMSS, viral evolution is expected to proceed quite differently from viral evolution in an admixed system at equilibrium. Mathematical modeling has provided many clinically useful insights into HIV dynamics; improved models are likely to give rise to improved insights.

## Methods

Here, we first present the set of ordinary differential equations on which the stochastic full metapopulation model is based. Because the SMSS solution of the full model cannot be found directly from the ordinary differential equations, we also derive a deterministic nested analytical approximation of the model (S1 Text) and study its equilibria (S1 Text).

### The Full Metapopulation Model

The full metapopulation model consists of a set of ordinary differential equations describing the number of susceptible cells *x*_*i*_(*t*), infected cells, *y*_*i*_(*t*), and HIV-specific CTLs *z*_*i*_(*t*) in each patch *i* at time *t*, as well as the number of infected cells in the blood, *y*_*B*_(*t*), and the number of latently infected long-lived resting CD4+ T cells, *L(t)*, which we refer to as the reservoir. We do not model the number of free virions and instead use the steady-state assumption that the number of free virions is proportional to the number of infected cells [16].

$$\frac{dx_{i}(t)}{dt}=\gamma_{i}\;M\;x_{B}-\frac{x_{i}(t)}{x_{i}^{max}}\;\beta_{i}\;y_{i}(t)-[d+\varepsilon ]\;x_{i}(t)$$ (Eq 1.1)

$$\frac{dy_{i}(t)}{dt}=(1-\lambda )\;\frac{x_{i}(t)}{x_{i}^{max}}\;\beta_{i}\;y_{i}(t)+\gamma_{i}[{M\;y}_{B}(t)+\omega \;a\;L(t)]-\left[\delta +\varepsilon +k\frac{z_{i}(t)}{z_{i}^{max}}\right]\;y_{i}(t)$$ (Eq 1.2)

$$\frac{dy_{B}(t)}{dt}=\sum_{j}\;\varepsilon \;y_{j}(t)-{M\;y}_{B}(t)-\delta_{B}\;y_{B}(t)$$ (Eq 1.3)

$$\frac{dz_{i}(t)}{dt}=c\;z_{i}^{max}\left[1-\frac{z_{i}(t)}{z_{i}^{max}}\right]-\varepsilon \;z_{i}(t)1_{y_{i}(t)=0}$$ (Eq 1.4)

$$\frac{dL(t)}{dt}=\sum_{i}\;\lambda \;\frac{x_{i}(t)}{x_{i}^{max}}\;\beta_{i}\;y_{i}(t)-(a+\delta_{L})\;L(t)$$ (Eq 1.5)

Eq 1.1 describes the dynamics of susceptible cells in patch *i*. Although CD4+ T cell count falls during the course of chronic infection, this decline is slow, particularly compared to the short durations of 100 days that we consider in our simulations, and at a good approximation the loss of CD4+ T cells is compensated for by their production through thymus-dependent and -independent pathways [61,62]. In addition, CTL and CD4+ T cell counts vary by a relatively small amounts among HIV-infected patients compared to the orders of magnitude differences in viral load [63]. We therefore assume that susceptible cells enter patches at a constant rate, *γ*_*i*_*M x*_*B*_, where *x*_*B*_ is the constant total number of susceptible cells in the blood, *M* is the rate at which cells leave the blood and enter patches, and *γ*_*i*_ is the probability that these cells enter patch *i* (∑_*i*_ *γ*_*i*_ = 1). Viral infectivity in patch *i* is given by *β*_*i*_, $x_{i}^{max}=\gamma_{i}M\;x_{B}/\left(d+\varepsilon \right)$ is the number of susceptible cells expected in patch *i* in the absence of infection, *d* is the death rate of susceptible cells, and *ε* is the rate at which lymphocytes exit patches.

In deriving the transmission term, we have assumed that patches have a fixed volume (proportional to $x_{i}^{max}$) and used the reasoning that virions released from an infected cell are more likely to reach other cells if the target cell population is more dense. Furthermore, once cells are infected by HIV, the CD4 is down-regulated on the cell surface, thus substantially reducing the probability of cell superinfection [64] and suggesting that free virions are only mildly “wasted” on already infected cells. Therefore, we assumed the number of susceptible cells infected by a single infected cell is $\beta_{i}\;x_{i}(t)/x_{i}^{max}$, which increases with the number (and density) of susceptible cells. The denominator $x_{i}^{max}$ appears explicitly, rather than being subsumed into *β*_*i*_, so that the value of *β*_*i*_ gives the maximum number of infections per day generated by a single infected cell.

Eqs 1.2 and 1.3 describe the dynamics of infected cells in patch *i* and in the blood, respectively. Here, *λ* is the proportion of cells that become long-lived upon infection and thus enter the reservoir, *a* is the activation rate of latently infected resting CT4+ T cells, *ω* is the proportion of activated cells that produce infectious virions, *δ* and *δ*_*B*_ are the death rates of infected cells within patches and in the blood, respectively, *k* is the maximum rate at which infected cells can be killed by CTLs (achieved when CTLs are at a maximum density within a patch), and $z_{i}^{max}$ is the maximum number of CTLs that patch *i* can accommodate. We assume that all patches are well connected, so that an infected cell egressing from a patch is as likely to enter a patch a long distance away as it is to enter an adjacent patch. Although this is clearly a simplification of the true trafficking pattern of infected cells [40], this is a reasonable first approximation, because significant migration of activated CD4+ T cells occurs over long ranges via the blood [36]. In addition, we also define the effective migration rate, *M*_*e*_, which is the rate at which infected cells leave a patch multiplied by the probability that an infected cell leaving a patch successfully enters a new patch, such that *M*_*e*_ = *ε M*/(*M* + *δ*_*B*_). Note that we assume that latently infected resting CD4+ T cells enter patches at random immediately upon reactivation. Although little is known about how resting CD4+ T cells circulate, their trafficking patterns are probably similar to other CD4+ T cells [36,65,66], and because these cells are long-lived, we can assume to a good approximation that they are randomly distributed among patches.

From experimental data, we know that HIV-specific CTLs accumulate at sites containing HIV-producing cells [48], although it is not clear whether CTL accumulation is due to increased rates of entry, e.g., [40], or lower rates of egress, e.g., [67–69]. In Eq 1.4, we assume that the maximum rate at which HIV-specific CTLs enter patches is *c z*^*max*^, with this rate gradually falling to zero as the patch reaches its maximum carrying capacity, *z*^*max*^; we impose a maximum carrying capacity on the patches, because otherwise the number of CTLs would grow without bound. The indicator function $1_{y_{i}(t)=0}$ takes the value 1 when *y*_*i*_*(t)* = 0 and 0 otherwise. Thus, HIV-specific CTLs are assumed to egress from patches at the same rate as CD4+ T cells, *ε*, if the patch contains no infected cells, but egress is prevented if infected cells are present [69]. We do not specifically model non-HIV-specific CTLs, because evidence suggests that only the egress of pathogen-specific CTLs is prevented in the presence of localized infection [69], and, thus, in the absence of co-infections, the number of non HIV-specific CTLs is expected to remain constant in all patches. Although we do not model the proliferation of HIV-specific CTLs within patches here, in the supplemental text we present a “CTL proliferation” version of the model in which this process is included. Since most CTL escape mutations probably sweep through the viral population during early infection [70–72], we also ignore the appearance of CTL escape mutations during chronic infection. Although such mutations do occur and sweep through within-host populations, they do so only slowly, suggesting their selective advantage is relatively weak [73].

Finally, Eq 1.5 describes the dynamics of the reservoir compartment, containing the set of latently infected resting CD4+ T cells, which die at rate *δ*_*L*_. A list of all of the parameters and their values is given in Table 1. This metapopulation model has some similarities to a recently published model of HIV infection in a network of lymphoid tissues [74], with the crucial difference that in our model local colonisation and extinction dynamics are observed due to the localised killing of infected cells as a consequence of the accumulation of CTLs within patches.

### Population-Based Simulation of the Full Metapopulation Model

We wrote a fully stochastic population-based simulation in C++ based on Eqs 1.1–1.5 and with a time step of 0.0001 to 0.001 days, depending on parameters (S1 Simulation Code). The number of events of each type (infection, death, and migration) occurring during a time step was drawn from either binomial or multinomial distributions as appropriate. We chose this simulation method rather than using Gillespie-type simulation to increase computational efficiency. Because this is intended to be a model of the chronic, asymptomatic phase of infection, all simulations were initiated with 10^8^ infected cells distributed evenly across the patches, so as to represent the large viral load associated with the end of the acute phase of infection [75]. We also assume that the number of HIV-specific CTLs has reached equilibrium at the systemic level (reflected in the constant parameter *c*) and that any CTL escape mutations have already swept through the viral population (enabling us to assume *k* is also constant), although the patches themselves are initiated with no HIV-specific CTL present. Because a large reservoir of long-lived latently infected cells is established during the early phases of infection [76], the simulations were initiated with a reservoir size of 10^7^, if included [44]. We introduced heterogeneity among the patches by sampling the values of *β*_*i*_ in patch *i* from the uniform distribution on [0.5 $\bar{\beta }$, 1.5 $\bar{\beta }$]. For all other parameters, the patches are assumed to be identical. Because for almost all parameter values the system reaches a steady state well before 40 days, the stationary number of infected cells was calculated by averaging the total number of infected cells in the metapopulation between days 60 and 100 from the start of the simulation. See S1 Data for the output from all the simulations.

### Single-Patch Model

We want to compare the results of the metapopulation model with the equivalent model, in which we have a single well-mixed tissue. The equations describing the single patch model are obtained by setting *N* = 1 and dropping index *i* from Eqs 1.1–1.5.

At equilibrium, the total number of infected cells is therefore given by:

$$Y_{tot}^{*}=max\;\left\{\frac{B}{\beta }\;[\frac{\beta (1-\lambda +\omega \;\lambda )-C}{C}](1+\frac{\varepsilon \;}{M+\delta_{B}}),0\right\}$$ (Eq 2)

where *C* = $\delta +\varepsilon \left(1-\frac{M}{M+\delta_{B}}\right)+k$. If the reservoir size is fixed, the equilibrium number of infected cells is instead given by:

$$Y_{tot}^{*}=\left(1+\frac{\varepsilon }{M+\delta_{B}}\right)\frac{1}{2\beta \;C}\left[B\;[\beta (1-\lambda )-C]+\omega \;a\;\beta \;\bar{L}+\sqrt{{\left(B\;[\beta (1-\lambda )-C]+\omega \;a\;\beta \;\bar{L}\right)}^{2}+4\;\omega \;a\;B\;\beta \;C\;\bar{L}}\right]$$ (Eq 3)

### Estimation of Parameters

Viral infectivity within patches, *β*, can be estimated from published values of *R*_0_ (the within-host basic reproduction number of HIV in the absence of a CTL response). Specifically, assuming infected cells spend the vast majority of the time within patches, *β* ≈ *δ R*_0_, where *δ* is the CTL-independent death rate of infected cells within patches. Since *δ* ≈ 1 per day [42,43], *β* ≈ *R*_0_. For HIV, estimates of *R*_0_ vary between 2 and 26 [77,78], giving us estimates of *β* between 2 and 26 per day. Noting that only two of the 51 patients included in these studies had an *R*_0_ higher than 20, and because in vitro measures of viral infectivity vary by more than a factor of 10 among patients [79], as does replicative capacity [80], taking a range of *β* values between 1 and 20 per day is likely to reasonably capture the heterogeneity among patients.

Estimating the maximum rate at which CTLs kill infected cells, *k*, is more problematical, with estimates in the literature ranging between 0.1 per day to 500 per day, depending on which methods and which viruses are used [81,82]. Estimates for HIV-1 range between 0.1 and 10 per day [81], but there is still considerable uncertainty as to how best to analyse available data, and particularly how the inclusion of high rates of CTL killing during the eclipse phase of the cell infection cycle (i.e., before viral production has started) alters the interpretation of the data [57]. In addition, we are interested in the maximum rate of CTL killing in very localized areas of the body, not the average rate of killing at the systemic level that is estimated from in vivo data, and therefore even the higher estimates for HIV-1 might underestimate *k*. We therefore take a range of *k* between 1 and 20 per day to reflect heterogeneity among individuals, with the caveat that it is important to recognize the uncertainties surrounding the choice of this range of values.

We interpret patches as the T cell areas in secondary lymphoid organs, such as lymph nodes, the spleen, and Peyer’s patches. Lymph nodes often contain several physically separated T cell areas [83], the number of which will depend on the size of the lymph node. In mice, there are about 1.6 T cell areas per lymph node on average [84]. Because the lymph nodes of humans are about 100 times heavier than those of mice, and there are 550 or so lymph nodes in adult humans, there are likely to be many thousands of patches in the lymph nodes of humans. In the spleen of a mouse there are about 10 Malpighian bodies, which are nodules of white pulp that contain many lymphocytes. Because a human spleen weighs about 1,500 times more than a mouse spleen, it also could easily contain thousands of patches. Finally, there are about 100–200 Peyer’s patches in a human gut [85]. Although by these rough calculations there could be, as an upper estimate, 100,000 patches in a human adult, in the main text we have used an estimate of *N* = 10,000, which is probably correct within an order of magnitude and makes the simulations computationally feasible. Because we do not yet have a full understanding of lymphocyte trafficking, patches might also be interpreted as larger areas within which there could be rapid movement of lymphocytes (a whole lymph node, for example). Therefore, we also investigate how the system behaves for *N* = 1,000 and *N* = 100 (see S1 Text).

To estimate the number of susceptible cells in the blood, *x*_*B*_, we first assume that the CD4+ T cell count in the blood of an HIV infected individual has fallen to a third of those in a healthy adult, giving a count of 333 cells per mm^3^. Although HIV is capable of infecting unactivated CD4+ T cells [86], we only consider replication in activated CD4+ T cells, since this is much more efficient. If we assume that 1% of CD4+ T cells are activated and a typical blood volume is 5 liters, this gives us *x*_*B*_ = 1.67 x 10^7^. The residence time of CD4+ T cells in the blood (including the vasculature of non-lymphoid tissue) is about 30 min [36], giving us a per capita migration rate, *M* = 48 per day.

Estimates from experimental data suggest that the residence time of lymphocytes in secondary lymphoid tissue is in the order of about 10 hours [39,40], and therefore we use a value of *ε* of 2.5 per day in our core parameters. In the spleen, residence time has been estimated to be shorter than this [39,40] giving a faster rate of egress from patches in the spleen. However, by our calculations, there are about 10 times more patches in the lymph nodes than in the spleen, and, in addition, a faster rate of egress will result in fewer infected cells; thus, the overall number of infected cells in the metapopulation will be driven by the longer duration patches.

To estimate the maximum number of HIV-specific CTLs per patch, we first note there are approximately 1x10^11^ CTLs in a typical human adult, many of which reside in secondary lymphoid tissue [24]. Since HIV-infected individuals have, on average, twice as many CTLs in the blood as uninfected individuals [87], and about 10% of these are HIV specific [88], then the average patch will have about 2x10^10^/*N* CTLs, where *N* is the number of patches. If the maximum carrying capacity of any individual patch is 5 times this, then a ballpark figure for *z*^*max*^ is 1x10^11^/*N*. Note that the within-patch dynamics depend on the CTL immigration parameter, *c*, and the density of CTLs in a patch ($z_{i}(t)/z_{i}^{max}$), not the absolute value of *z*^*max*^. We use a value of *c* = 0.5, as this means the number of CTLs within a patch typically reaches a maximum in between 1 and 4 days, in line with empirical observations [89]. Using these values, we can estimate the number of HIV-specific CTLs expected in an uninfected patch, $z_{0}=c\;z_{i}^{max}/\left(c+\varepsilon \right)$ = 1.67 x 10^6^, if *N* = 10,000 and all patches are identical.

All other parameters are estimated directly from the literature (see Table 1).

## Supporting Information

S1 AnimationAn example of the dynamics of the metapopulation at shifting mosaic steady state for 100 of the 10,000 patches simulated.The simulated metapopulation was initialised with 10^8^ infected cells randomly distributed among the patches, and the animation spans 60 days (day 40 to day 100 of the simulation). The coloured discs inside each circle represent the Log_10_ number of infected cells in each of the patches, with the colour getting darker and the radius getting larger as the Log_10_ number of infected cells increases. The maximum number of infected cells in any of the patches is approximately 10^3^. Any patches with fewer than 10 infected cells are kept white, because otherwise the constant “flicking” of the cells between white and pale yellow is very distracting. We assume all patches are well connected due to migration via the blood, so patches that are in close proximity in the animation are equally connected as those that are a distance apart. The patches experiencing the biggest bursts of infection are characterised by the largest values of β in [0.5 $\bar{\beta }$, 1.5 $\bar{\beta }$] = [5,15], and no patches reach a steady state. Although there are oscillations in the total number of infected cells, which are apparent when watching the animation, these are relatively minor at the scale of the whole metapopulation (see S1 Fig). We have assumed a high effective migration rate (*M*_*e*_ = 2.4 per day), no reservoir, $\bar{\beta }$ *= 10* per day, and *k = 13* per day. All other parameters are as in Table 1.(MP4)

S1 DataOutput data from the metapopulation simulations.Each file gives the output from the simulations indicated by the filename: low migration (ms1, db432); high migration (ms1, db1); very high migration (ms10, db1); number of patches (np); reservoir (storage); no reservoir (nostorage); CTL immigration model (CTLimmig); CTL proliferation model (CTLprolif); $\bar{\beta }$ (beta1, beta2, …). Within each file, there are 21 simulations for (*k* = 0,1,..,20), and data is output each day. For measures of synchrony, the final entry should be used. See Methods and S1 Simulation Code for further details.(ZIP)

S2 DataLikelihood calculations given observed frequencies of SPVL among seroconverters in the Netherlands (S1 Table).Each row in the files gives the likelihood of the model (for specified $\bar{\beta }$, standard deviation of $\bar{\beta }$ among patients, mean *k*, standard deviation of *k* among patients). The three files represent the 10,000 patch CTL immigration model; the 10,000 patch CTL proliferation model; and the single patch model. A reservoir is assumed to be present in all cases. All other parameters are as in Table 1. See S1 Text for further details.(ZIP)

S1 FigSteady state for the metapopulation simulation in the absence of a reservoir for different numbers of patches.Total number of infected cells, synchrony among patches, and amplitude of oscillations are shown. The blue density plots in the top row show the number of infected cells at steady state (note that mean viral infectivity varies across patches, and therefore mean viral infectivity $\bar{\beta }=\beta$ is plotted). The areas in white are where no infected cells are present. The green density plots in the second row give a measure of synchrony among patches, measured between days 60 and 100, where 1 is completely synchronised and 0 is no synchrony (see S1 Text). The high levels of synchrony found towards the origin arise because the system has not reached steady state by day 60, and therefore do not reflect oscillatory behavior for these parameters. The red density plots in the third row show the relative amplitude of oscillations in the Log_10_ number of infected cells at steady state, where 1 means the amplitude of the oscillations is equal to the maximum number of infected cells measured, and 0 means the number of infected cells remains constant (see S1 Text). For all of the density plots, the area between the black solid lines shows where an SMSS is observed, defined as where both the viral population has not gone extinct and the mean CTL response across all patches is less than 99% of the maximum CTL response, *k*. The bottom two rows are transects across the density plots shown above them, with either viral infectivity held constant (*β* = 10 per day) or the maximum strength of the CTL response held constant (*k* = 10 per day). Number of infected cells, blue-scale gradient and blue dots. Synchrony, green-scale gradient and green dots. Amplitude, red-scale gradient and red dots. Simulations were initiated with 10^8^ infected cells. We assume a high effective migration rate (*M*_*e*_ = 2.4 per day). All other parameters are as described in Table 1.(PDF)

S2 Fig Steady state for the metapopulation simulation in the presence of a reservoir for different numbers of patches. Similar to S1 Fig except the simulations were initialized with a reservoir of 10^7^ latently infected resting CD4+ T cells. All other parameters are as described in Table 1. Note that in the presence of a reservoir the virus cannot go extinct. The high relative amplitude found at low viral loads is a consequence of infecting cells stochastically leaving the reservoir. As the number of patches decreases, the effect of this stochasticity becomes more pronounced.
(PDF)

S3 Fig Steady state for the metapopulation simulation in the absence of a reservoir for different effective migration rates. Similar to S1 Fig except the number of patches is always 10,000 and instead the effective migration rate, *M*_*e*_, is varied. Low *M*_*e*_ = 0.25 per day, high *M*_*e*_ = 2.4 per day, very high *M*_*e*_ = 25 per day. All other parameters are as described in Table 1.
(PDF)

S4 Fig Steady state for the metapopulation simulation in the presence of a reservoir for different effective migration rates. Similar to S1 Fig, except the simulations were initialized with a reservoir of 10^7^ latently infected resting CD4+ T cells, the number of infected cells is always 10,000, and instead the effective migration rate, *M*_*e*_ is varied. Low *M*_*e*_ = 0.25 per day, high *M*_*e*_ = 2.4 per day, very high *M*_*e*_ = 25 per day. All other parameters are as described in Table 1. Note that in the presence of a reservoir the virus cannot go extinct.
(PDF)

S5 Fig Examples of within-patch dynamics for a single colonization event used for the analytical solution of the CTL proliferation model. Maximum strength of the CTL response, *k = 5* per day. Viral infectivity *β* (per day) *=* 10, black; 9, purple; 8, blue; 7, cyan; 6, green; 5, orange; 4, brown. For a description of the CTL proliferation model see S1 Text. All parameters are as described in Table 1, except *c* = 0.001 per day, and *g*, a measure of CTL proliferation, equals 1 per day, and with *ε* = 2.5 per day.
(PDF)

S6 Fig Steady state for the metapopulation simulation of the CTL proliferation model in the absence of a reservoir and for different numbers of patches. Similar to S1 Fig except *c* = 0.001 per day, and *g*, a measure of CTL proliferation, equals 1 per day. For a description of the CTL proliferation model see S1 Text. All other parameters are as described in Table 1.
(PDF)

S7 Fig Steady state for the metapopulation simulation of the CTL proliferation model in the presence of a reservoir and for different numbers of patches. Similar to S1 Fig except *c* = 0.001 per day, *g*, a measure of CTL proliferation, equals 1 per day, and the simulations were initialized with a reservoir of 10^7^ latently infected resting CD4+ T cells. For a description of the CTL proliferation model see S1 Text. All other parameters are as described in Table 1.
(PDF)

S8 Fig Steady state for the metapopulation simulation of the CTL proliferation model in the absence of a reservoir and for different effective migration rates. Similar to S1 Fig except *c* = 0.001 per day, *g*, a measure of CTL proliferation, equals 1 per day, the number of patches is always 10,000, and instead the effective migration rate, *M*_*e*_ is varied. Low *M*_*e*_ = 0.25 per day, high *M*_*e*_ = 2.4 per day, very high *M*_*e*_ = 25 per day. For a description of the CTL proliferation model see S1 Text. All other parameters are as described in Table 1.
(PDF)

S9 Fig Steady state for the metapopulation simulation of the CTL proliferation model in the presence of a reservoir and for different effective migration rates. Similar to S1 Fig except *c* = 0.001 per day, *g*, a measure of CTL proliferation, equals 1 per day, the simulations were initialized with a reservoir of 10^7^ latently infected resting CD4+ T cells, the number of infected cells is always 10,000, and instead the effective migration rate, *M*_*e*_ is varied. Low *M*_*e*_ = 0.25 per day, high *M*_*e*_ = 2.4 per day, very high *M*_*e*_ = 25 per day. For a description of the CTL proliferation model see S1 Text. All other parameters are as described in Table 1. Note that in the presence of a reservoir the virus cannot go extinct.
(PDF)

S10 FigMaximum log-likelihood distributions of set-point viral load for the Single Patch, 10,000 patch CTL Immigration, and 10,000 patch CTL Proliferation models and the bivariate marginal likelihood surfaces given the Netherlands seroconverter data.The CTL Immigration model is the standard metapopulation model used throughout the manuscript, by which CTLs accumulate within patches due to immigration. The CTL proliferation model is a variant of this model, in which CTLs accumulate due to local proliferation within patches (see S1 Text). Viral infectivity, *β*, and the maximum CTL immune response, *k*, are assumed to be distributed according to truncated normal distributions, with probability density functions *f* (*β*; *μ*_*β*_,*σ*_*β*_,*β*_*min*_,*β*_*max*_) and *f* (*k*; *μ*_*k*_,*σ*_*k*_,*k*_*min*_,*k*_*max*_), respectively, where *β*_*min*_ = 1.05 per day, *k*_*min*_ = 0 per day, and *β*_*max*_ = *k*_*max*_ = 20 per day (see S1 Text). Single Patch model, maximum log $L(\mu_{\beta }=14,\sigma_{\beta }=4,\mu_{k}=15,\sigma_{k}=3|Data)$ = -5,386. CTL Immigration model maximum log $L(\mu_{\beta }=14,\sigma_{\beta }=4,\mu_{k}=15,\sigma_{k}=3|Data)$ = -4,013. CTL Proliferation model maximum log $L(\mu_{\beta }=11,\sigma_{\beta }=1,\mu_{k}=12,\sigma_{k}=2|Data)$ = -3,768. For all models, the viral reservoir is assumed to be present and the effective migration rate, *M*_*e*_, is assumed to be high (*M*_*e*_ = 2.4 per day). All other parameters are as described in the Table 1.(PDF)

S1 Simulation Code C++ code for the metapopulation simulations. (CPP)

S1 Table Observed set-point viral loads among seroconverters in the Netherlands. We thank Stichting HIV Monitoring for sharing this data.
(CSV)

S1 Text Supplementary methods. (PDF)

## Abbreviations

ART: antiretroviral therapy
CTL: cytotoxic T lymphocyte
FE: full equilibrium
LN: lymph node
SIV: simian immunodeficiency virus
SMSS: shifting-mosaic steady state
SPVL: set-point viral load
