# Supplementary figures and images for: Large Variations in HIV-1 Viral Load Explained by Shifting-Mosaic Metapopulation Dynamics
Source: PLoS Biol. 2016 Oct 5;14(10):e1002567. doi: 10.1371/journal.pbio.1002567 (PMC5051940; doi:10.1371/journal.pbio.1002567)

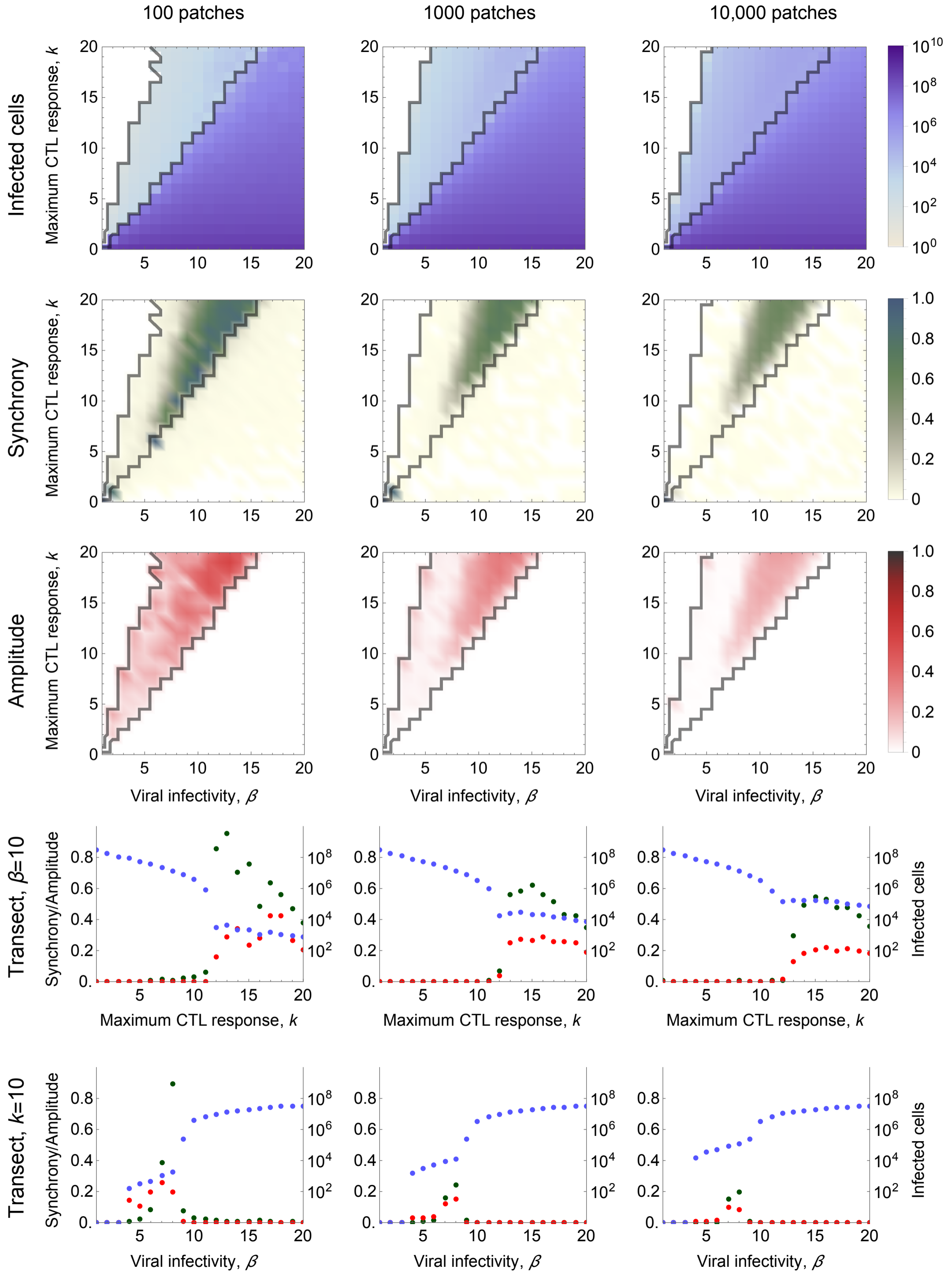

Supplement: S1 Fig — Total number of infected cells, synchrony among patches, and amplitude of oscillations are shown. The blue density plots in the top row show the number of infected cells at steady state (note that mean viral infectivity varies across patches, and therefore mean viral infectivity β¯=β is plotted). The areas in white are where no infected cells are present. The green density plots in the second row give a measure of synchrony among patches, measured between days 60 and 100, where 1 is completely synchronised and 0 is no synchrony (see S1 Text). The high levels of synchrony found towards the origin arise because the system has not reached steady state by day 60, and therefore do not reflect oscillatory behavior for these parameters. The red density plots in the third row show the relative amplitude of oscillations in the Log10 number of infected cells at steady state, where 1 means the amplitude of the oscillations is equal to the maximum number of infected cells measured, and 0 means the number of infected cells remains constant (see S1 Text). For all of the density plots, the area between the black solid lines shows where an SMSS is observed, defined as where both the viral population has not gone extinct and the mean CTL response across all patches is less than 99% of the maximum CTL response, k. The bottom two rows are transects across the density plots shown above them, with either viral infectivity held constant (β = 10 per day) or the maximum strength of the CTL response held constant (k = 10 per day). Number of infected cells, blue-scale gradient and blue dots. Synchrony, green-scale gradient and green dots. Amplitude, red-scale gradient and red dots. Simulations were initiated with 108 infected cells. We assume a high effective migration rate (Me = 2.4 per day). All other parameters are as described in Table 1. (PDF) [file pbio.1002567.s004.pdf]

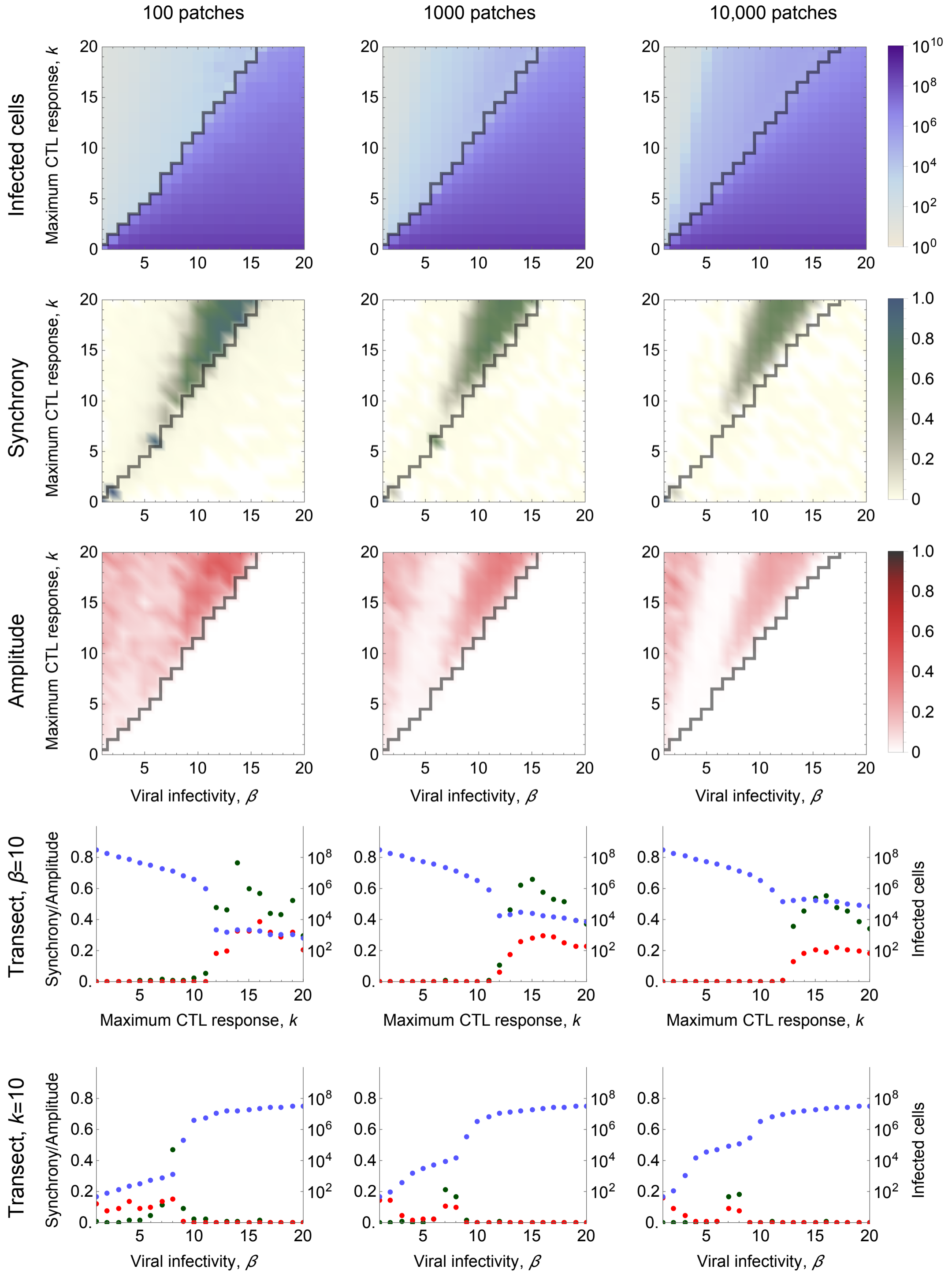

Supplement: S2 Fig — Similar to S1 Fig except the simulations were initialized with a reservoir of 107 latently infected resting CD4+ T cells. All other parameters are as described in Table 1. Note that in the presence of a reservoir the virus cannot go extinct. The high relative amplitude found at low viral loads is a consequence of infecting cells stochastically leaving the reservoir. As the number of patches decreases, the effect of this stochasticity becomes more pronounced. (PDF) [file pbio.1002567.s005.pdf]

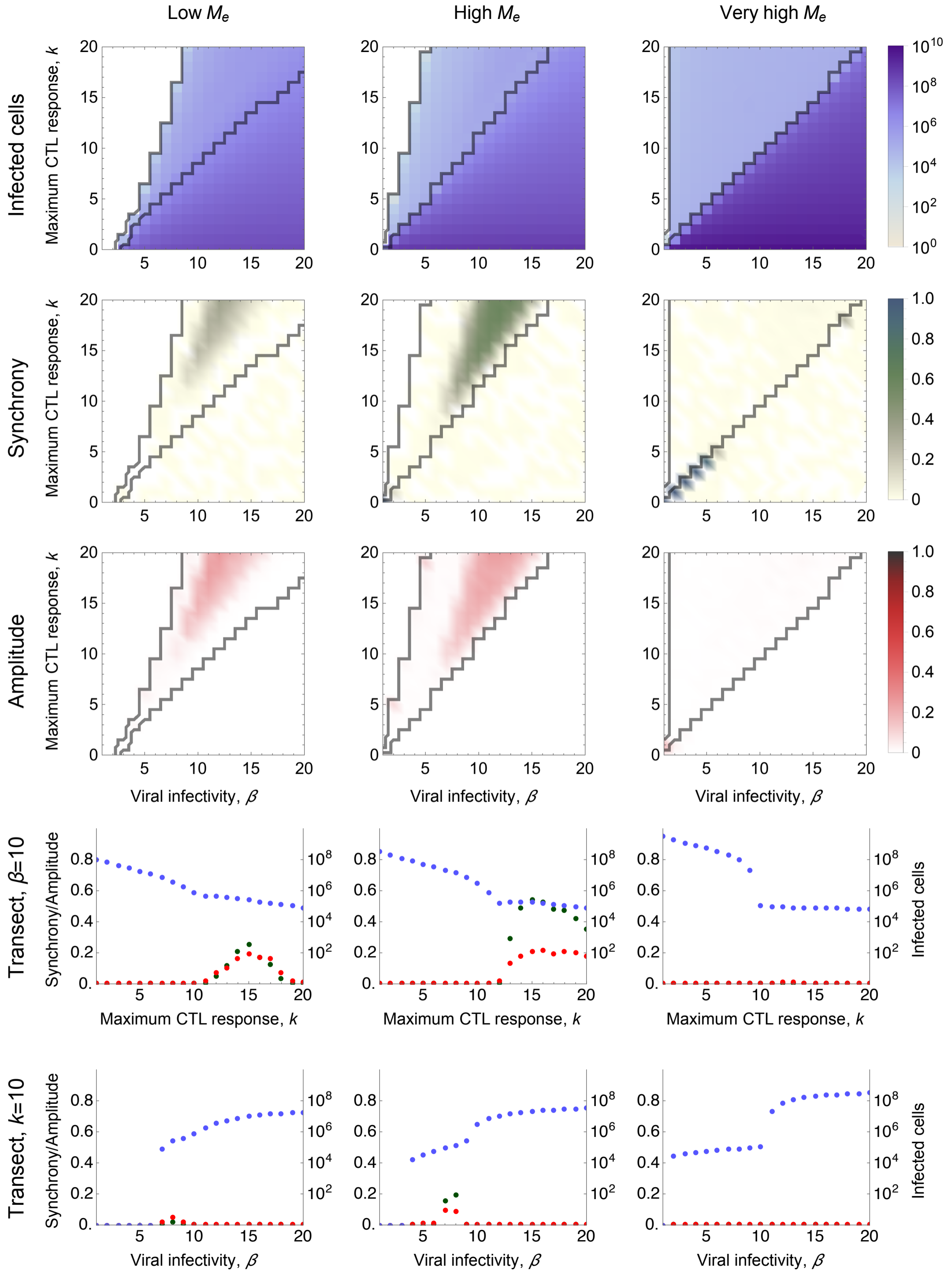

Supplement: S3 Fig — Similar to S1 Fig except the number of patches is always 10,000 and instead the effective migration rate, Me, is varied. Low Me = 0.25 per day, high Me = 2.4 per day, very high Me = 25 per day. All other parameters are as described in Table 1. (PDF) [file pbio.1002567.s006.pdf]

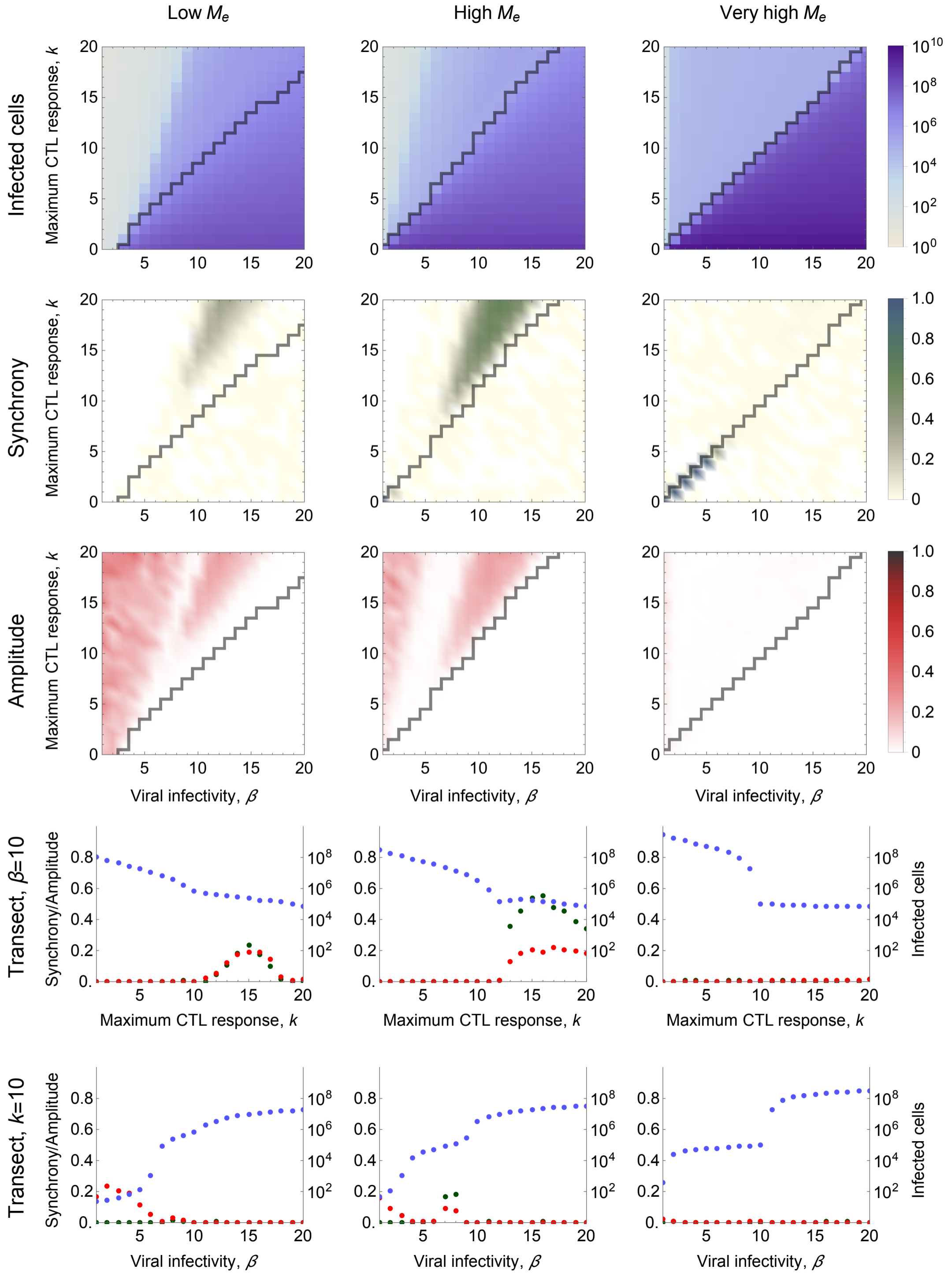

Supplement: S4 Fig — Similar to S1 Fig, except the simulations were initialized with a reservoir of 107 latently infected resting CD4+ T cells, the number of infected cells is always 10,000, and instead the effective migration rate, Me is varied. Low Me = 0.25 per day, high Me = 2.4 per day, very high Me = 25 per day. All other parameters are as described in Table 1. Note that in the presence of a reservoir the virus cannot go extinct. (PDF) [file pbio.1002567.s007.pdf]

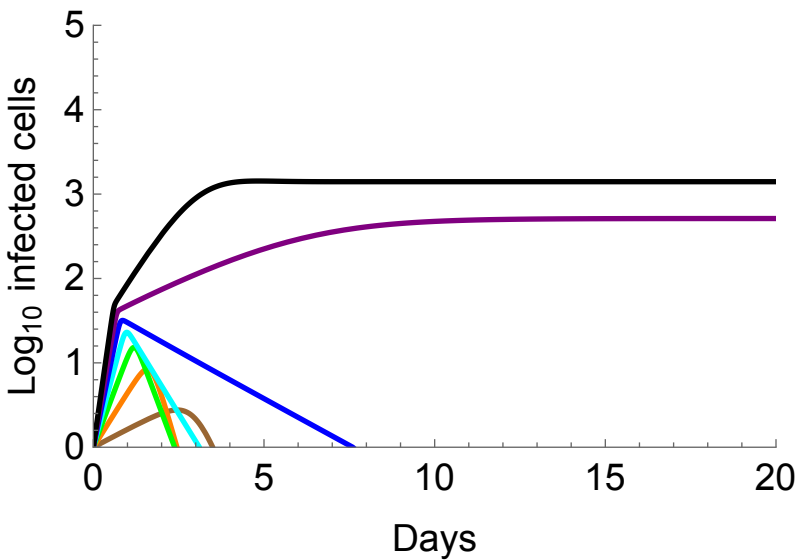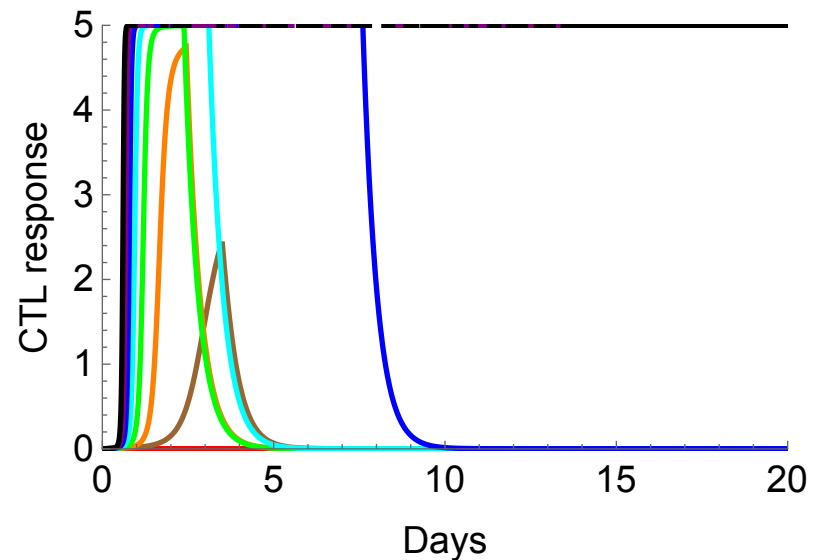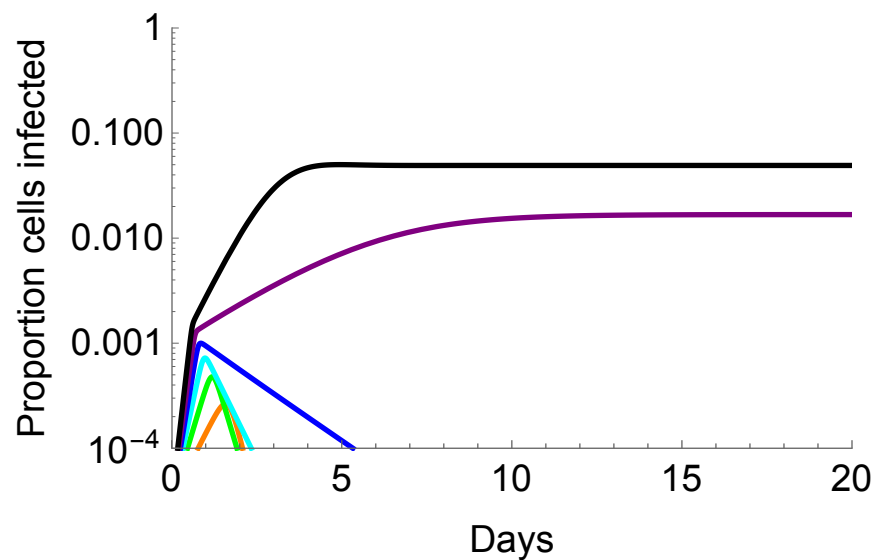

Supplement: S5 Fig — Maximum strength of the CTL response, k = 5 per day. Viral infectivity β (per day) = 10, black; 9, purple; 8, blue; 7, cyan; 6, green; 5, orange; 4, brown. For a description of the CTL proliferation model see S1 Text. All parameters are as described in Table 1, except c = 0.001 per day, and g, a measure of CTL proliferation, equals 1 per day, and with ε = 2.5 per day. (PDF) [file pbio.1002567.s008.pdf]

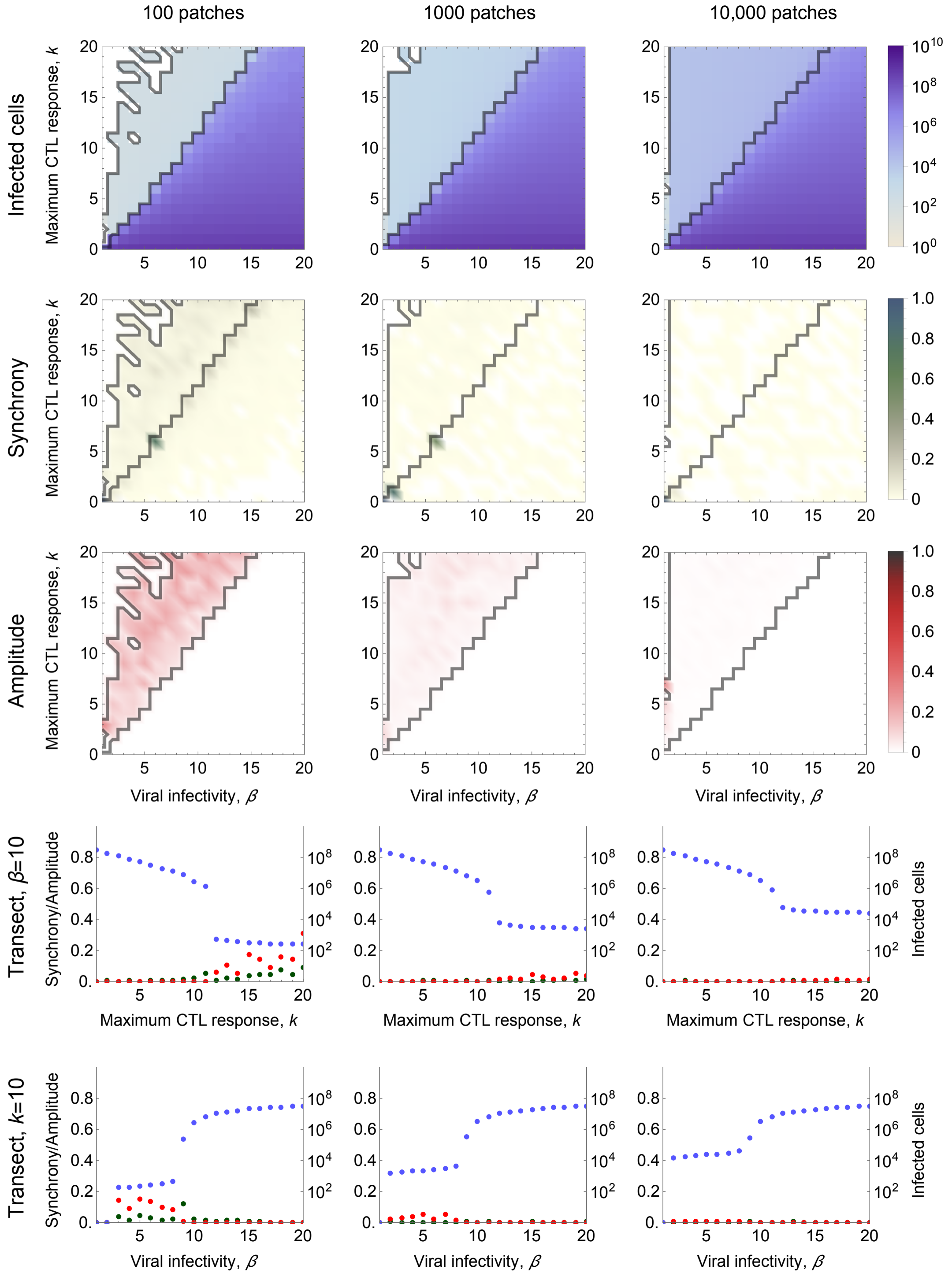

Supplement: S6 Fig — Similar to S1 Fig except c = 0.001 per day, and g, a measure of CTL proliferation, equals 1 per day. For a description of the CTL proliferation model see S1 Text. All other parameters are as described in Table 1. (PDF) [file pbio.1002567.s009.pdf]

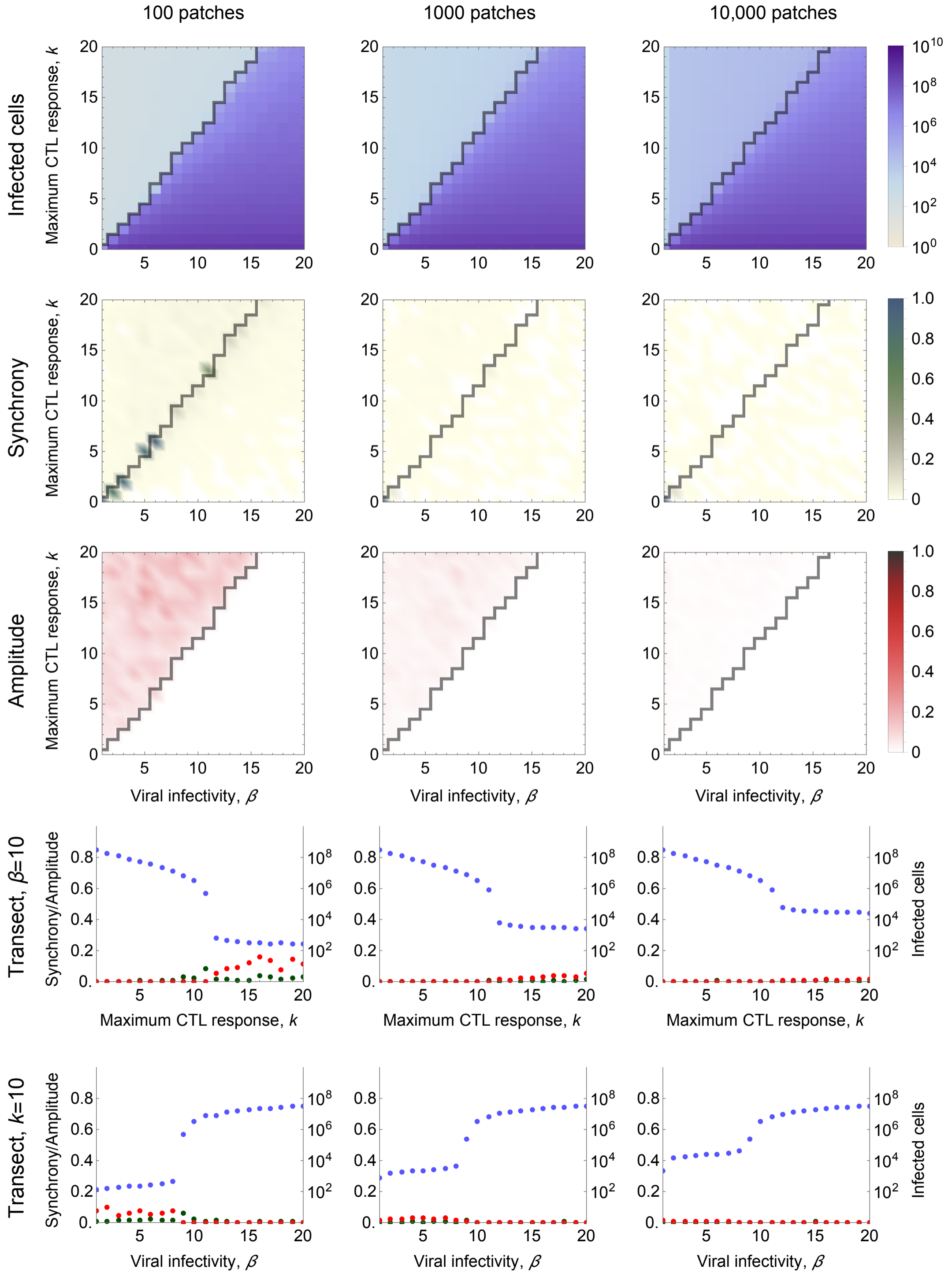

Supplement: S7 Fig — Similar to S1 Fig except c = 0.001 per day, g, a measure of CTL proliferation, equals 1 per day, and the simulations were initialized with a reservoir of 107 latently infected resting CD4+ T cells. For a description of the CTL proliferation model see S1 Text. All other parameters are as described in Table 1. (PDF) [file pbio.1002567.s010.pdf]

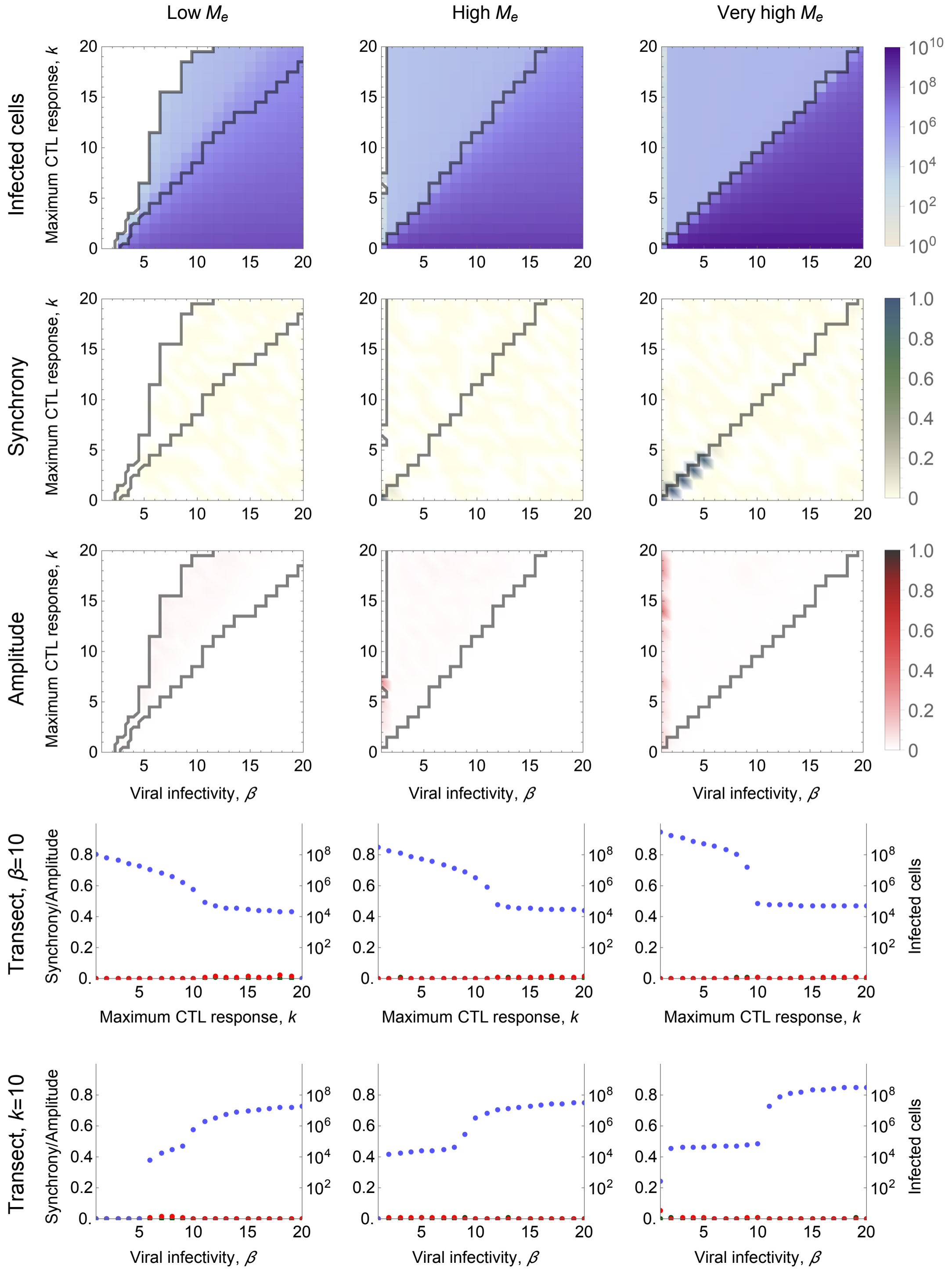

Supplement: S8 Fig — Similar to S1 Fig except c = 0.001 per day, g, a measure of CTL proliferation, equals 1 per day, the number of patches is always 10,000, and instead the effective migration rate, Me is varied. Low Me = 0.25 per day, high Me = 2.4 per day, very high Me = 25 per day. For a description of the CTL proliferation model see S1 Text. All other parameters are as described in Table 1. (PDF) [file pbio.1002567.s011.pdf]

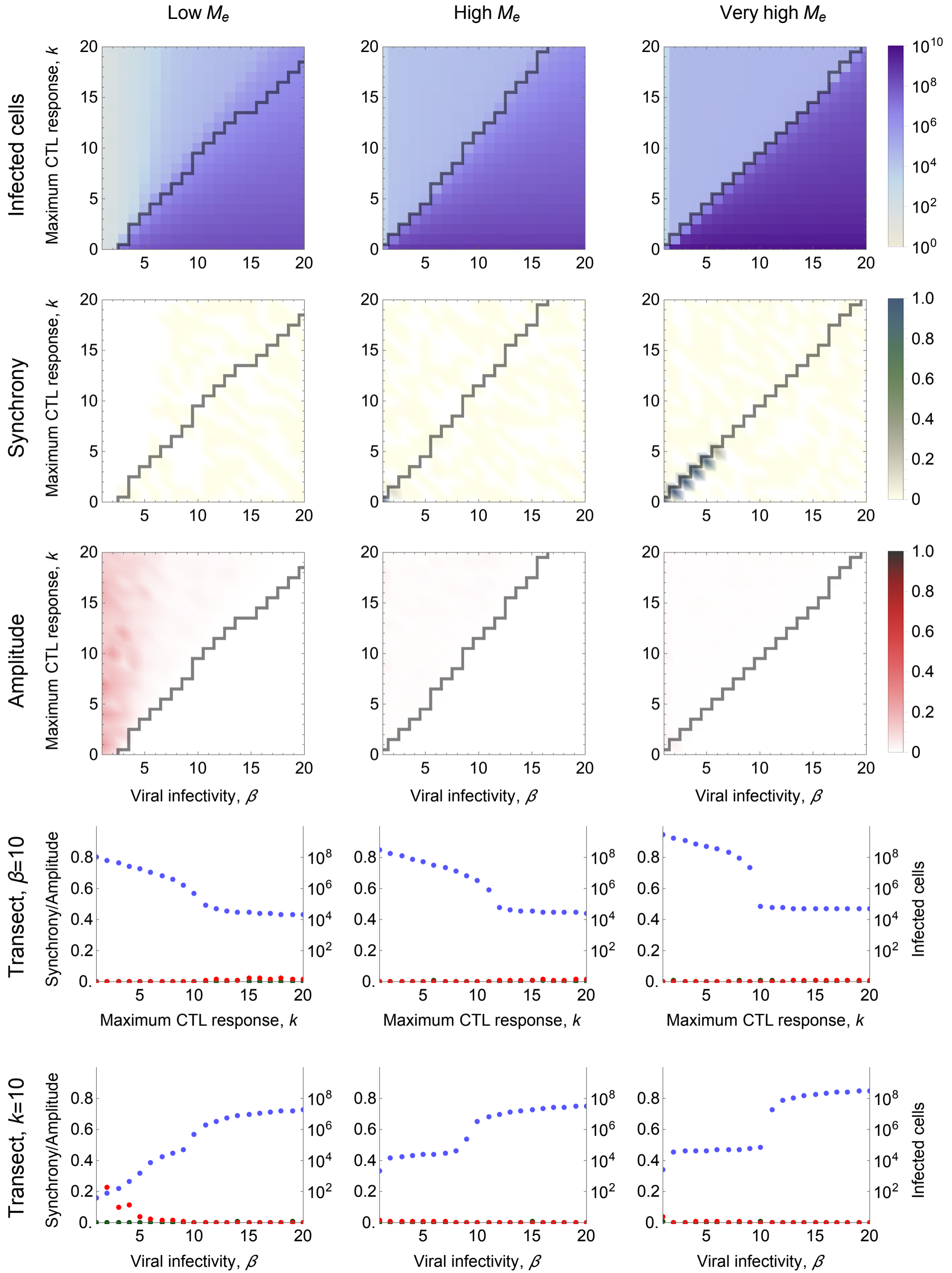

Supplement: S9 Fig — Similar to S1 Fig except c = 0.001 per day, g, a measure of CTL proliferation, equals 1 per day, the simulations were initialized with a reservoir of 107 latently infected resting CD4+ T cells, the number of infected cells is always 10,000, and instead the effective migration rate, Me is varied. Low Me = 0.25 per day, high Me = 2.4 per day, very high Me = 25 per day. For a description of the CTL proliferation model see S1 Text. All other parameters are as described in Table 1. Note that in the presence of a reservoir the virus cannot go extinct. (PDF) [file pbio.1002567.s012.pdf]

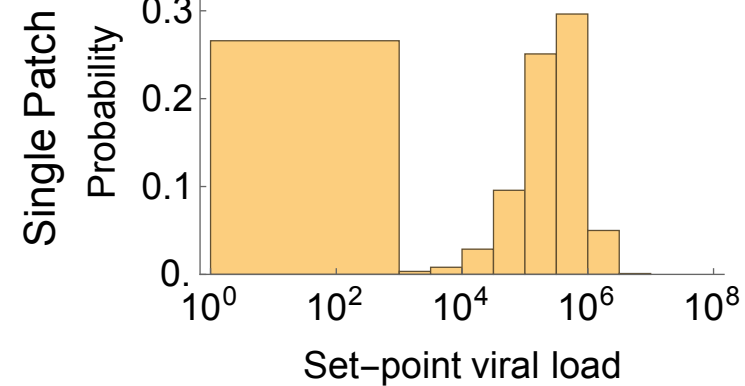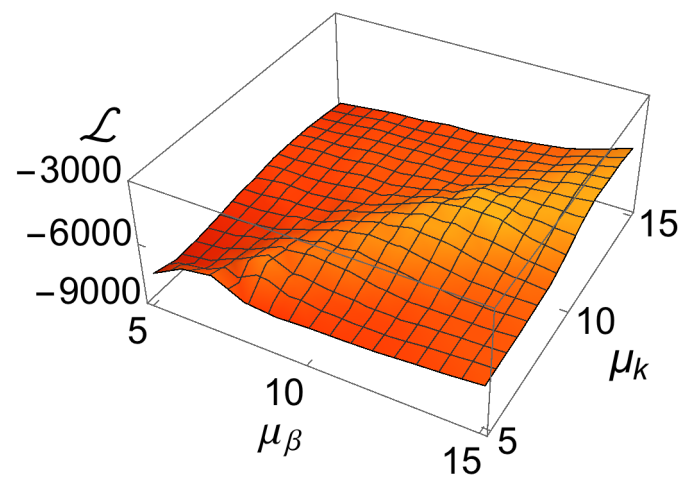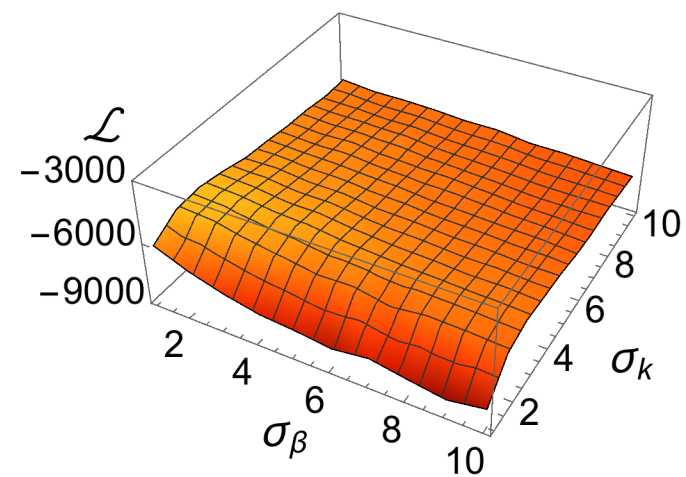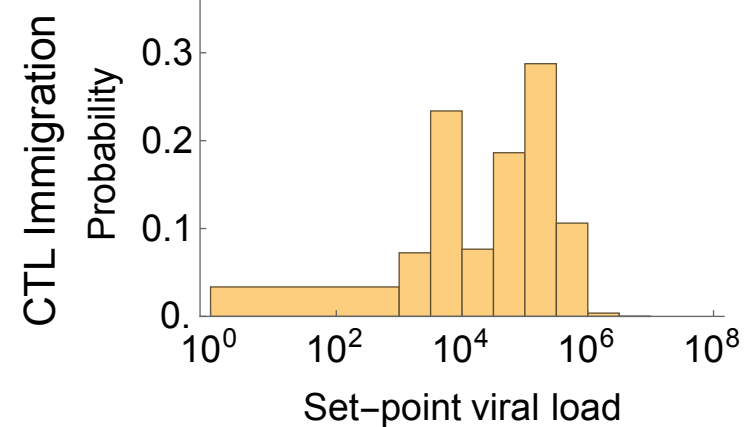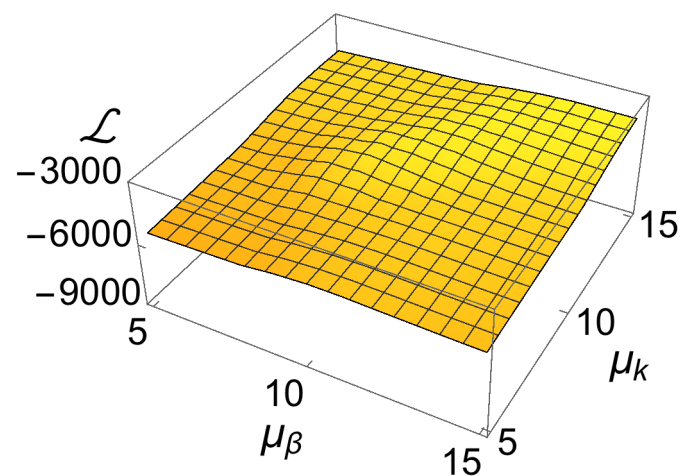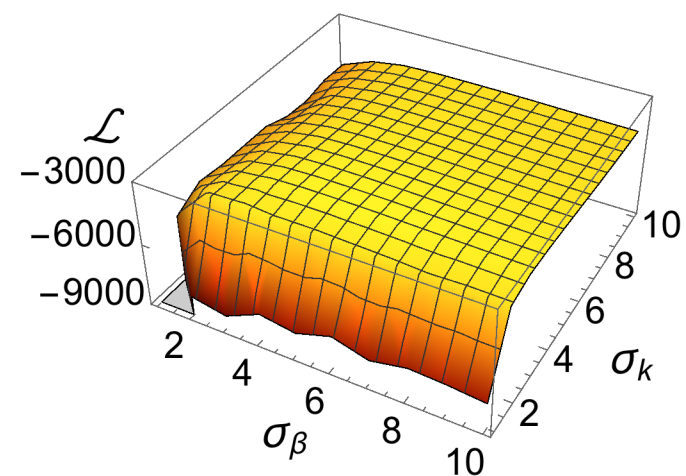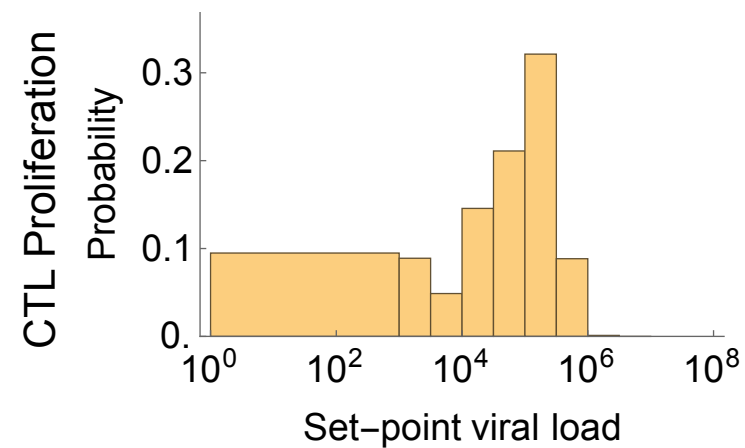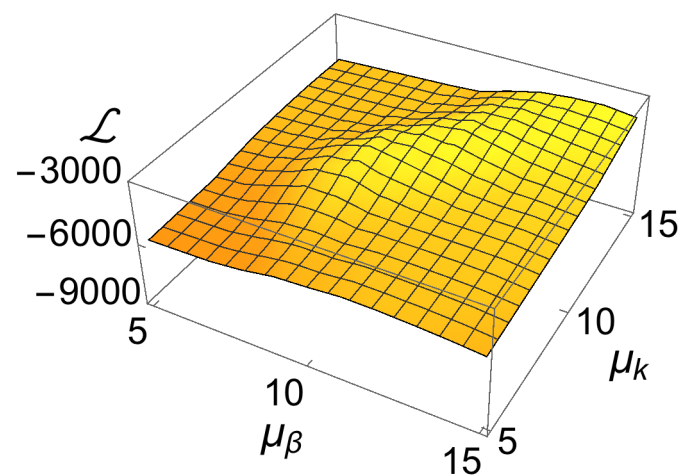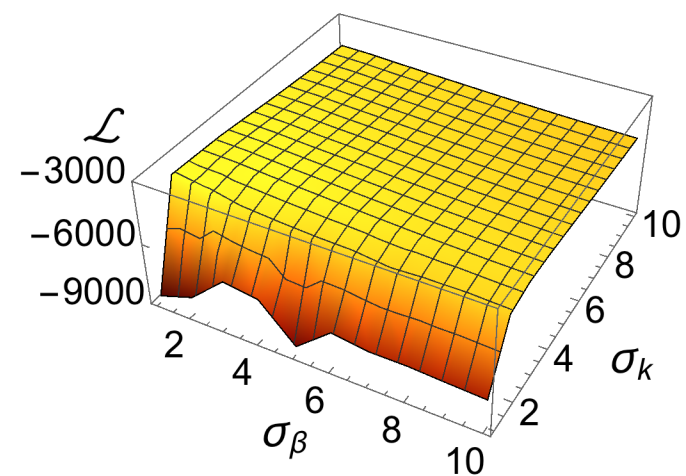

Supplement: S10 Fig — The CTL Immigration model is the standard metapopulation model used throughout the manuscript, by which CTLs accumulate within patches due to immigration. The CTL proliferation model is a variant of this model, in which CTLs accumulate due to local proliferation within patches (see S1 Text). Viral infectivity, β, and the maximum CTL immune response, k, are assumed to be distributed according to truncated normal distributions, with probability density functions f (β; μβ,σβ,βmin,βmax) and f (k; μk,σk,kmin,kmax), respectively, where βmin = 1.05 per day, kmin = 0 per day, and βmax = kmax = 20 per day (see S1 Text). Single Patch model, maximum log L(μβ=14,σβ=4,μk=15,σk=3|Data) = -5,386. CTL Immigration model maximum log L(μβ=14,σβ=4,μk=15,σk=3|Data) = -4,013. CTL Proliferation model maximum log L(μβ=11,σβ=1,μk=12,σk=2|Data) = -3,768. For all models, the viral reservoir is assumed to be present and the effective migration rate, Me, is assumed to be high (Me = 2.4 per day). All other parameters are as described in the Table 1. (PDF) [file pbio.1002567.s013.pdf]
