# Supplementary material for: Large Variations in HIV-1 Viral Load Explained by Shifting-Mosaic Metapopulation Dynamics
Source: PLoS Biol. 2016 Oct 5;14(10):e1002567. doi: 10.1371/journal.pbio.1002567 (PMC5051940; doi:10.1371/journal.pbio.1002567)
Supplement: S1 Text — (PDF) [file pbio.1002567.s016.pdf]

Large Variations in HIV-1 Viral Load Explained by Shifting-Mosaic Metapopulation Dynamics

Supplementary Methods

Katrina A. Lythgoe[1,2]*, François Blanquart[2], Lorenzo Pellis[3], Christophe Fraser[2,4]

1 Department of Zoology, Tinbergen Building, University of Oxford, Oxford, United Kingdom,
2 Department of Infectious Disease Epidemiology, School of Public Health, Imperial College London, St. Mary's Campus, London, United Kingdom,
3 Mathematics Institute, Zeeman Building, University of Warwick, Coventry, United Kingdom,
4 Big Data Institute, Li Ka Shing Centre for Health Information and Discovery, Nuffield Department of Medicine, University of Oxford, Oxford, United Kingdom

* katrina.lythgoe@zoo.ox.ac.uk

1. Analytical approximation of the metapopulation model
2. Sensitivity of total number of infected cells to the effective rate of migration and the number of patches
3. Calculation of synchrony and relative amplitude
4. The "CTL-proliferation" model
5. Likelihood calculations and optimization

1. Analytical approximation of the metapopulation model

Here we detail how we derived the analytical approximation of the metapopulation model, by first considering the within-patch dynamics and then nesting these within-patch dynamics in a metapopulation framework.

Nested analytical approximation: within-patch dynamics

To derive the deterministic analytical approximation of the full metapopulation model, we first make the simplifying assumption that all patches are identical, and therefore $\gamma_i = 1/N \ \forall i$. Because the influx of infected cells into a patch from the latent reservoir is small (see Table 1), if the influx rate of infected cells into a patch from the blood is also sufficiently small, once a patch has been colonized by infected cells we can neglect the effects of further entry of infected cells on the

subsequent within-patch dynamics. The within-patch dynamics can now be described by the following set of equations, where τ is the time since a patch was initially colonized:

$$\frac{dx(\tau)}{d\tau} = \frac{M\, x_B}{N} - \frac{x(\tau)}{x^{max}}\, \beta\, y(\tau) - [d + \varepsilon]\, x(\tau) \qquad \text{(S1.1)}$$

$$\frac{dy(\tau)}{d\tau} = (1-\lambda)\, \frac{x(\tau)}{x^{max}}\, \beta\, y(\tau) - \left[\delta + \varepsilon + k\, \frac{z(\tau)}{z^{max}}\right] y(\tau) \qquad \text{(S1.2)}$$

$$\frac{dz(\tau)}{d\tau} = c\, z^{max} \left[1 - \frac{z(\tau)}{z^{max}}\right] - \varepsilon\, z(\tau)\, \mathbf{1}_{y(\tau)=0} \qquad \text{(S1.3)}$$

Assuming the patch contains no infected cells and is at equilibrium before colonization, initial conditions are given by the disease free equilibrium $x(0) = x^{max}$, $y(0) = 0$, and $z(0) = c\, z^{max}/(c + \varepsilon)$. Upon colonization by a single infected cell, $y(0) \to 1$. In a deterministic framework the number of infected cells cannot fall to zero, so we impose the condition that if the number of infected cells falls below one then no further infection can take place and that egress of CTLs from the patch resumes. The within-patch dynamics described by Eqs S1.1-S1.3 exhibit one of three qualitatively distinct outcomes (i) a within-patch burst of infection fails to establish; (ii) a short-lived burst of infection followed by local extinction of infected cells; or (iii) a burst of infection followed by the establishment of a stable endemic state (Fig 3).

Based on Eqs S1.1 – S1.3, a within-patch burst of infection will fail to establish (outcome (i)) if the reproduction number of the virus when the patch is initially colonized, $R_{init} = \beta\,(1-\lambda)/\left(\delta + \varepsilon + k\, \frac{c}{(c+\varepsilon)}\right)$, is less than 1. In the presence of infected cells, the number of CTLs within a patch will monotonically increase until the maximum number of CTLs, z^{max}, is reached. A non-zero endemic state will be established (outcome (iii)) if the reproduction number of the virus when the strength of the immune response within the patch is at a maximum, $R_{endemic} = \beta\,(1-\lambda)/(\delta + \varepsilon + k)$ is greater than or equal to 1. In all other cases, a short-lived epidemic will occur (outcome (ii)).

Nested analytical approximation: metapopulation dynamics

Next, we nest the within-patch dynamics within a metapopulation framework using a next-generation approach, an established approach in epidemiological modeling [1–3], so that we can describe the dynamics of the metapopulation as a whole.

The first step is to write an ordinary differential equation for the number of infected cells in the blood at time t:

$$\frac{dy_B(t)}{dt} = \varepsilon \, \mathrm{Y(t)} - (M + \delta_B)\, y_B(t) \qquad \text{(S2.1)}$$

where $Y(t)$ is the total number of infected cells summed across all the patches. We can also write an equation for the size of the reservoir, *L(t)*:

$$\frac{dL(t)}{dt} = \sum_i \lambda \, \frac{x_i(t)}{x^{max}} \, \beta \, y_i(t) - (\rho + \delta_L)\, L(t) \qquad \text{(S2.2)}$$

The next step is to find an expression for $Y(t)$. Assume that, of the total of N patches, at time *t, C(t)* are colonized by infected cells and/or an excess of CTLs, and the rest, *U(t)=N-C(t)*, are uncolonised. If we define *H(t)* as the rate at which empty patches are colonised at time t (this is analogous to incidence in epidemiological models), then we can write an expression for $Y(t)$:

$$Y(t) = \int_0^{\infty} H(t-\tau)\, y(\tau)\, \psi(\tau) d\tau \qquad \text{(S2.3)}$$

where $\psi(\tau)$ is the probability that a patch is still colonized τ days since its initial colonization. Eq. S2.3 states that the total number of infected cells now (time t) results from the contribution of all patches colonized τ units of time before, $H(t-\tau)$, and which therefore now contain $y(\tau)$ infected cells (provided the patches are still colonized, i.e. with probability $\psi(\tau)$). All contributions are added by integrating over all times τ from now ($\tau = 0$) back to the time of the initial conditions (τ=t) and before. In theory, unlike the full metapopulation model for which initial conditions are all defined at time $t = 0$, the nested model would require specifying the history of $H(t), t < 0$, as part of the initial conditions. However, we refrain from doing so explicitly here because we are interested in identifying the system's equilibria, which do not depend on the initial conditions. In the deterministic model the duration of colonization is fixed and constant, a function of only the parameters of the model. However, the deterministic model is intended as an approximation to stochastic dynamics. To include the impact of stochastic variation and patch heterogeneity, we included the parameter $\psi(\tau)$, and specifically we assume the duration of infection is gamma distributed with mean duration *T* and shape parameter 10 (variance = T^2/10). Without including heterogeneity in this way we found that patches synchronise and the system can exhibit stable limit cycles, and we chose the parameter 10 since this minimised any synchronisation effect. This synchronisation effect was also observed for the simulations (Figs 2, S1-S4). The duration *T* is calculated from forward integration of Eqs S1.1 – S1.3, and is the time it takes for the number of infected cells within a patch to fall below 1 and, in addition, for the strength of the immune response to fall to within 1% of the within-patch steady state immune response in the absence of infection.

Finally, $H(t)$ is given by:

$$H(t) = \frac{U(t)}{N}\big(M\, y_B(t) + \omega\, a\, L(t)\big) \qquad \text{(S2.4)}$$

with $U(t) = N - C(t) = N - \int_{\tau=0}^{\infty} H(t-\tau)\, \psi(\tau) d\tau$.

We are ultimately interested in the equilibrium number of infected cells in the nested model, since this is our proxy for SPVL. Using a star to denote values at equilibrium (i.e. $H(t) = H^* \;\forall t$ at equilibrium), Eq. S2.3 gives us the equilibrium number of infected cells summed across all the patches:

$$Y^* = H^* \int_0^{\infty} y(\tau)\psi(\tau) d\tau. \qquad \text{(S3.1)}$$

Using Eq. S2.1 and Eq. S3.1, we can then find the equilibrium number of infected cells in the blood:

$$y_B^* = \frac{\varepsilon\, Y^*}{(M + \delta_B)} \qquad \text{(S3.2)}$$

which finally allows us to write an expression for the total number of infected cells in the metapopulation as a whole at equilibrium:

$$Y_{tot}^* = Y^* + y_B^* = Y^*\left(1 + \frac{\varepsilon}{M + \delta_B}\right) \qquad \text{(S3.3)}$$

We also note that the rate of patch colonization at equilibrium, H^*, is 0 if $R_P = R_B + R_L \leq 1$, or is given by:

$$H^* = \frac{N}{T}\left(1 - \frac{1}{R_P}\right) \quad if\ R_P > 1 \qquad \text{(S3.4)}$$

Here, R_P is the "patch-to-patch reproduction number", which is defined as the number of patches that a single patch infects during a single colonization event when all other patches are uncolonised. R_P is the sum of $R_B = \int_{\tau=0}^{\infty} \frac{M}{(M+\delta_B)}\, \varepsilon\, y(\tau)\psi(\tau) d\tau$, which is the contribution to R_P from infected cells transiting via the blood, where $M/(M + \delta_B)$ is the proportion of infected cells egressing from a patch that successfully reach a new patch when transiting through the blood, and $R_L = \int_{\tau=0}^{\infty} \frac{a}{(a+\delta_L)}\omega\, \lambda\, \frac{\mathrm{x}(t)}{x^{max}}\, \beta\, y(\tau)\psi(\tau) d\tau$, which is the contribution to R_P from infected cells transiting via the reservoir compartment, where $a/(a + \delta_L)$ is the proportion of cells entering the reservoir that are reactivated.

We have so far assumed that we have a "dynamic reservoir" that can change in size, and therefore if the rate at which cells enter the reservoir is less than the rate at which they are activated, the equilibrium reservoir size will be zero. However, because the rate of reactivation, *a,* and the death rate of cells in the reservoir, δ_L, are both very small, any depletion in the size of the reservoir will be extremely slow, and much slower than the timescales of a couple of hundred days that we are considering here. Since the reservoir is established early in infection [4,5] and then maintained in the long term even when individuals are put onto antiretroviral therapy [5–7], we make the further assumption for the analytical approximation (but not the simulations) that the reservoir is of a fixed constant size throughout infection; that is, $L(t) = \bar{L}\ \forall t$. Without this assumption, the equilibrium reservoir size will be zero, even though this will be approached long after the time-scale that we are interested in. This implicitly assumes that the reservoir is maintained due to the proliferation and/or death of infected cells [8,9]. With a non-zero constant size reservoir, no disease-free equilibrium is possible; even in the absence of transmission from patch to patch ($R_B = 0$) the rate of patch colonization at equilibrium is $H^*_{fixed} = \omega\ a\ \bar{L}$ and, for $R_B > 0$, it is now given by:

$$H^*_{fixed} = \frac{1}{2T\ R_B}\left(N\ (R_B - 1) - \omega\ a\ T\bar{L} + \sqrt{(N\ (R_B - 1) - \omega\ a\ T\bar{L})^2 + 4\ \omega\ a\ \bar{L}\ N\ T\ R_B}\right) \quad \text{(S4)}$$

If the colonization of patches is short-lived, the equilibrium of the nested metapopulation model represents a SMSS, where the number of infected cells as a whole will be at equilibrium, but where the patches will not be (Eqs S3.1-S3.4). Alternatively, if patches reach an endemic state of infection, the equilibrium represents a FE, where the number of infected cells within all of the patches will be at a non-zero equilibrium. If this condition is satisfied, Y^*_{tot}, can be written simply as:

$$Y^*_{tot} = N\ y^*\left(1 + \frac{\varepsilon}{M + \delta_B}\right) = \frac{M\ x_B}{\beta}\left[\frac{\beta(1-\lambda) - (\delta + \varepsilon + k)}{\delta + \varepsilon + k}\right]\left(1 + \frac{\varepsilon}{M + \delta_B}\right) \quad \text{(S5)}$$

where y^* is the equilibrium number of infected cells within a patch, which can be computed directly from Eqs S1.1-S1.3 and does not depend on the reservoir (whether fixed or dynamic), as the immigration during a single-patch burst is assumed negligible in the nested approximation.

2. Sensitivity of total number of infected cells to the effective migration rate and the number of patches

The analytical approximation to the metapopulation model is expected to be accurate only when the rate at which infected cells enter patches is very low, so that once a patch is infected the within-patch dynamics are unaffected by the further entry of infected cells into the patch. To quantify the rate of immigration in the model, we define the "effective" migration rate as the rate at which infected cells egress from patches multiplied by the probability that these cells successfully reach a new patch:

$$M_e = \frac{\varepsilon M}{M+\delta_B}$$

Using the parameters in Table 1, this gives us a "high" effective migration rate of $M_e \approx 2.4$ per day. To obtain a "low" M_e more inline with the assumption of the analytical approximation we assume only 10% of infected cells entering the blood reach a new patch by setting $\delta_B = 432$ per day, giving $M_e \approx 0.25$ per day. Although a value of $\delta_B = 432$ per day is very high, our understanding of lymphocyte trafficking is still incomplete and therefore such a high value might not be unreasonable. Infected CD4+ T cells probably have decreased motility compared to uninfected cells [10], and infected cells entering patches may well be killed by an effective CTL response before an appreciable number of virions are produced. Nonetheless, given the available evidence, we deem high M_e values more realistic.

We also consider a "very high" effective migration rate scenario by setting $\varepsilon = 25$ per day and $M = 480$ per day, giving us $M_e \approx 25$ per day. Although such high migration rates are unrealistic for HIV, they enable us to gain additional insight into how the model behaves when there is a high degree of mixing of infected cells among patches.

Although the analytical approximation is expected to be accurate in the low migration scenario, comparisons with the population based stochastic simulations for different M_e reveal that the quality of the approximation remains satisfactory even for high M_e values (Fig 4). This suggests that, at least for most values of β, the immigration of infected cells once a burst has been initiated has little effect on the overall within-patch dynamics – a key assumption of the nested approximation.

At very high levels of M_e we might expect the simulations to begin to behave like the single-patch well-mixed model. However, we still see the persistence of a (relatively small) number of infected cells for most values of β and k (S3 Fig),

rather than the extinction scenario we would expect from the single-patch model (Fig 4). This apparently paradoxical result can be explained as follows: At low M_e, reintroductions of infected cells into empty patches are relatively rare, leaving sufficient time for a large proportion of CTLs to egress between bursts of infection, which in turn results in large numbers of infected cells accumulating during a burst before the immune system has the time to mount up. As M_e increases, migration tends to erode large bursts and redistribute the cells more evenly among patches, and hence CTLs will accumulate in patches because they are only rarely, if ever, uncolonised, which in turn will drive down the number of infected cells. However, once the number of infected cells is driven down to about the same order as the number of patches, some patches will not contain any infected cells. Due to the very rapid egress of CTLs from empty patches, these patches will also contain a reduced number of CTLs making them available once more for successful colonisation; it is this process that maintains a small population of infected cells in the metapopulation. If we decrease the number of patches, fewer infected cells can be maintained in the SMSS regime and the closer the metapopulation model results resemble those of the single-patch model (S1 Fig).

3. Calculation of synchrony and relative amplitude

Oscillations are sometimes observed in the total number of infected cells in the metapopulation when at SMSS. To investigate these oscillations we calculated both the synchrony among patches and the relative amplitude of the oscillations. Synchrony is the temporal correlation in the number of infected cells between two patches, averaged over all possible pairs of patches [11]. Because the possible number of pairings is very large, we estimated synchrony by taking the average correlation in the number of infected cells between days 60 and 100 of 2500 pairs of patches (all possible pairs between patches 0-49 and 50-99). Synchrony values can range from -1 to 1, where -1 means all pairs of patches are perfectly in antiphase, 0 means the phases are independent across patches, and 1 means all patches are perfectly in phase. In practice, values of synchrony <0 were rare, and only marginally less than zero, and so we only plot values of synchrony between 0 and 1 (S1-S4 Figs).

Despite the caricature of constant viral load during chronic infection, measurements taken from individuals can vary considerably between time points, although typically not by orders of magnitude. We therefore also calculated the relative amplitude of oscillations in the number of infected cells as:

$\frac{\log_{10} Y^{max} - \log_{10} Y^{min}}{\log_{10} Y^{max}}$

where Y^{max} is the maximum number of infected cells observed during days 60-100 of the simulations, and Y^{min} is the minimum. Measurements of both synchrony and relative amplitude for the metapopulation model are shown in S1-S4 Figs.

Appreciable oscillations among patches are only observed when the metapopulation is at SMSS and when there are more than 15-20 infected cells per patch, averaged over all patches. In addition, the level of synchrony dips as the system approaches FE because some patches will be at equilibrium whilst others will not be. As might be expected, levels of synchrony increase as the number patches decreases. However, levels of synchrony tend to be highest when the effective migration rate, M_e, is high. When M_e is low, only 10% of infected cells egressing from a patch successfully reach a new patch, thus limiting the ability of patches to synchonise. At very high M_e, infected cells rapidly circulate among patches (CD4+ T cells remain in a patch for less than an hour on average), which reduces the size of bursts of infection, thus preventing synchronisation among patches. In the presence of a reservoir, we also see reasonably large fluctuations in the number of infected cells when viral loads are very low due to the activation of latently infected resting CD4+ T cells.

4. The "CTL-proliferation" model

In the main text we assume CTLs accumulate within patches because egress from these patches is prevented, whilst immigration of CTLs continues (we call this the "CTL-immigration" model). Alternatively, CTLs might accumulate as a result of local proliferation due to the presence of antigen. To investigate how the local proliferation of CTLs affects our model results, we developed a "CTL-proliferation model". This is identical to the "CTL-immigration" model, except Eq. 1.4 is replaced with:

$$\frac{dz_i(t)}{dt} = [c\, z_i^{max} + g\, y_i(t)\, z_i(t)]\left[1 - \frac{z_i(t)}{z_i^{max}}\right] - \varepsilon\, z_i(t)\mathbf{1}_{y_i(t)=0} \qquad \text{(S6)}$$

where g is a measure of the CTL proliferation rate. For the CTL proliferation model we use values of c = 0.001 per day, and g = 1 per day. As with the CTL immigration model, we chose these parameters so that the number of CTLs within a patch typically reaches its maximum in between 1 and 4 days, in line with empirical observations [12].

Results for the CTL proliferation model are shown in Figures S5-S10. A key difference between the CTL proliferation model compared to the CTL immigration model is that the rate at which CTLs accumulate in a patch depends on how rapidly the number of infected cells increases (S5 Fig). In particular, CTLs proliferate more rapidly as the number of infected cells in the patch increases, thus limiting the size of bursts of infection. When the metapopulation is at a SMSS we therefore see more moderate increases in the number of infected cells as β increases or k decreases (compared to the CTL immigration model) followed by a large jump in the number of infected cells as the system moves from SMSS to FE (S6-S9 Figs). In addition, due to the small burst size, we do not see the synchronisation among patches that we observe for the CTL immigration model and oscillations in the total number of infected cells (S6-S9 Figs). Because at SMSS bursts of infection tend to be small in size, at low migration rates the metapopulation is prone to stochastic extinction for parameters where the nested approximation predicts a SMSS should be sustained. We do not see this for the CTL immigration model due to the larger bursts of infection in this model.

5. Likelihood calculations and optimization

We calculated the likelihood of different distributions of set-point viral load (SPVL) given the single patch model and the "CTL immigration" and "CTL proliferation" metapopulation models, with a high effective migration rate, M_e = 2.4 per day, and in the presence of a reservoir. The SPVLs for the single patch model were derived using the analytical solution to the single patch model, whilst the SPVLs for the metapopulation model were derived from simulations. We assume that β and k are distributed according to truncated normal distributions with probability density functions $f(\beta;\ \mu_\beta, \sigma_\beta, \beta_{min}, \beta_{max})$ and $f(k;\ \mu_k, \sigma_k, k_{min}, k_{max})$ respectively, independent of each other. Since an infection is not expected to establish in the first place if the reproduction number of infected cells at the time of infection (i.e. in the absence of a CTL response and of a reservoir), R_{noCTL}, is less than 1, we can calculate β_{min} as:

$$\beta_{min} = \frac{\delta + \varepsilon\left(1 - \frac{M}{M+\delta_B}\right)}{1-\lambda} \approx 1.05$$

where the term in parentheses accounts for the fact that, of all infected cells that egress from patches, a fraction $\frac{M}{M+\delta_B}$ will re-enter patches (see Table 1 for parameters). We further assume k_{min}= 0 and $\beta_{max} = k_{max}$ = 20, since these are the limits used in our simulations. We calculated the log-likelihood of the metapopulation model and the single patch model given the Dutch data (S1 Table), for all integer values of μ_β, μ_k between 5 and 15, and integer values of σ_β,

σ_k between 1 and 10. Because of differences in the sensitivities of viral load tests, we pooled all individuals in the Dutch cohort with a viral load less than 10^3 per ml, and for all other individuals we pooled the viral data into half log increments (i.e. 10^3-$10^{3.5}$, $10^{3.5}$-10^4, etc.) to give the number of individuals in each bin, n_i. Denoting by $\phi(\nu_i|\mu_\beta, \sigma_\beta, \mu_k, \sigma_k)$ the probability that the SPVL (computed from the simulation) of an individual with β and k drawn from the truncated Normal distributions with parameters $\mu_\beta, \sigma_\beta, \mu_k, \sigma_k$ falls in bin i, and assuming the Dutch cohort is representative of the set of people in chronic HIV infection stage, so that individuals in the cohort are distributed in the bins according to a multinomial distribution, the log-likelihood is proportional to:

$$\log \mathcal{L}(\mu_\beta, \sigma_\beta, \mu_k, \sigma_k|Data) \propto \sum_i n_i \log \left(\phi(\nu_i|\mu_\beta, \sigma_\beta, \mu_k, \sigma_k)\right)$$

The method for calculating the log-likelihood for the single-patch model was similar, except we randomly selected ten thousand paired values of β and k (not necessarily integer) from the truncated normal distributions, and used the equilibrium of the single patch model directly to calculate the expected SPVL for each of these paired values of β and k. The predicted maximum likelihood distributions of SPVL for the CTL immigration model, the CTL proliferation model and the single patch model are shown in S10 Fig, together with the corresponding bivariate marginal likelihood profiles. All likelihood values are reported in S2 Data.

References

1. Diekmann O, Heesterbeek JAP. Mathematical Epidemiology of Infectious Diseases: Model Building, Analysis and Interpretation. Wiley series in mathematical and computational biology. Wiley; 2000.
2. Lythgoe KA, Pellis L, Fraser C. Is HIV short-sighted? Insights from a multistrain nested model. Evolution (N Y). 2013;67: 2769–82. doi:10.1111/evo.12166
3. Sasaki A, Iwasa Y. Optimal growth schedule of pathogens within a host: switching between lytic and latent cycles. Theor Popul Biol. 1991;39: 201–239.
4. Chun T-W, Engel D, Berrey MM, Shea T, Corey L, Fauci AS. Early establishment of a pool of latently infected, resting CD4+ T cells during primary HIV-1 infection. Proc Natl Acad Sci U S A. 1998;95: 8869–73.
5. Whitney JB, Hill AL, Sanisetty S, Penaloza-MacMaster P, Liu J, Shetty M, et al. Rapid seeding of the viral reservoir prior to SIV viraemia in rhesus monkeys. Nature. Nature Publishing Group; 2014;512: 74–77. doi:10.1038/nature13594

6. Siliciano JD, Kajdas J, Finzi D, Quinn TC, Chadwick K, Margolick JB, et al. Long-term follow-up studies confirm the stability of the latent reservoir for HIV-1 in resting CD4+ T cells. Nat Med. 2003;9: 727–8. doi:10.1038/nm880

7. Chun T-W, Fauci AS. HIV reservoirs: pathogenesis and obstacles to viral eradication and cure. AIDS. 2012;26: 1261–8. doi:10.1097/QAD.0b013e328353f3f1

8. Chomont N, El-Far M, Ancuta P, Trautmann L, Procopio FA, Yassine-Diab B, et al. HIV reservoir size and persistence are driven by T cell survival and homeostatic proliferation. Nat Med. 2009;15: 893–900. doi:10.1038/nm.1972

9. Maldarelli F, Wu X, Su L, Simonetti FR, Shao W, Hill S, et al. Specific HIV integration sites are linked to clonal expansion and persistence of infected cells. Science. 2014;345: 179–183. doi:10.1126/science.1254194

10. Murooka TT, Deruaz M, Marangoni F, Vrbanac VD, Seung E, von Andrian UH, et al. HIV-infected T cells are migratory vehicles for viral dissemination. Nature. Nature Publishing Group; 2012;490: 283–287. doi:10.1038/nature11398

11. Rock K, Brand S, Moir J, Keelling MJ. Dynamics of Infectious Diseases. Reports Prog Phys. 2014;77: 026602. doi:10.1088/0034-4885/77/2/026602

12. Veiga-Fernandes H, Walter U, Bourgeois C, McLean a, Rocha B. Response of naïve and memory CD8+ T cells to antigen stimulation in vivo. Nat Immunol. 2000;1: 47–53. doi:10.1038/76907
